# Single-Point Linkage Engineering in Conjugated Phthalocyanine-Based Covalent Organic Frameworks for Electrochemical CO_2_ Reduction

**DOI:** 10.1007/s40820-025-01754-9

**Published:** 2025-05-09

**Authors:** Wenchang Chen, Yi Zhang, Mingyu Yang, Chao Yang, Zheng Meng

**Affiliations:** Department of Chemistry, University of Science and Technology of China, Hefei, 230026 Anhui People’s Republic of China

**Keywords:** Conjugated covalent organic frameworks, Linkage engineering, Single heteroatom tuning, CO_2_ electroreduction

## Abstract

The utilization of covalent organic frameworks (COFs) holds great potential for achieving tailorable tuning of catalytic performance through bottom-up modulation of the reticular structure. In this work, we show that a single-point structural alteration in the linkage within a nickel phthalocyanine (NiPc)-based series effectively modulates the catalytic performance of the COFs in electrochemical CO_2_ reduction reaction (CO_2_RR). A NiPc-based COF series with three members which possess the same NiPc unit but different linkages, including piperazine, dioxin, and dithiine, have been constructed by nucleophilic aromatic substitution reaction between octafluorophthalocyanine nickel and tetrasubstituted benzene linkers with different bridging groups. Among these COFs, the dioxin-linked COF showed the best activity of CO_2_RR with a current density of CO (*j*_CO_) =  − 27.99 mA cm^−2^ at − 1.0 V (versus reversible hydrogen electrode, RHE), while the COF with piperazine linkage demonstrated an excellent selectivity of Faradaic efficiency for CO (FE_CO_) up to 90.7% at a pretty low overpotential of 0.39 V. In addition, both a high FE_CO_ value close to 100% and a reasonable *j*_CO_ of − 8.20 mA cm^–2^ at the potential of − 0.8 V (versus RHE) were obtained by the piperazine-linked COF, making it one of the most competitive candidates among COF-based materials. Mechanistic studies exhibited that single-point structural alteration could tailor the electron density in Ni sites and alter the interaction between the active sites and the key intermediates adsorbed and desorbed, thereby tuning the electrochemical performance during CO_2_RR process.

**Supplementary Information:**

The online version contains supplementary material available at 10.1007/s40820-025-01754-9.

## Highlights

- Three novel covalent organic frameworks (COFs) composed of nickel phthalocyanine units and different linkages, including dioxin, piperazine, and dithiine, were successfully constructed.
- It was found that only a single-point structural variation of the linkage in the COFs could effectively modulate their performance in CO_2_ reduction reaction, where the piperazine-linked COF achieved a pretty high Faradaic efficiency for CO of 90.7% at a critically low overpotential of 0.39 V.
- Theoretical calculations indicated that the COF with dioxin linkage stabilized the *COOH intermediate more effectively than the other two NiPc-based COFs.

**Supplementary Information:**

The online version contains supplementary material available at 10.1007/s40820-025-01754-9.

## Graphical abstract

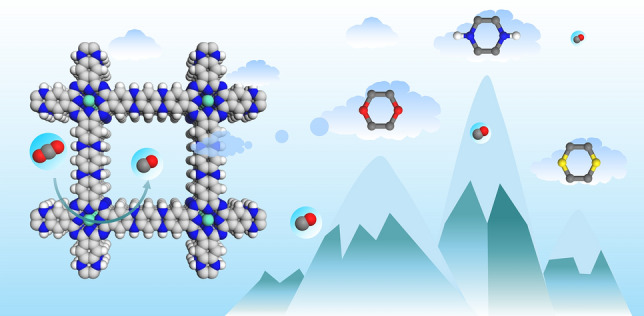

## Introduction

Electrochemical carbon dioxide reduction reaction (CO_2_RR) represents an effective pathway to mitigate the increasing concentration of atmospheric CO_2_; meanwhile, it produces value-added products [1, 2]. Over the years, a variety of homogeneous and heterogeneous catalysts have been developed to improve the activity, selectivity, and energy efficiency of electrochemical CO_2_RR. Homogeneous catalysts, exemplified by a few metal coordination complexes, including metalloporphyrins (MPys) [3, 4], metallophthalocynines (MPcs) [5, 6], and their derivates [7, 8], often have highly versatile and tailorable structures to give high selectivity [9, 10], however, with low catalytic activities due to the hindered charge transfer. By contrast, heterogeneous materials [11], such as widely applied metal oxides [12], graphene [13], and polymers [14], usually demonstrate high efficiency and activity due to their inherent high conductivity [15]. However, these materials often have difficulty in offering precise atomic-level control over the activity and selectivity. The quest for novel catalysts that integrate high activity, high selectivity, and tunability within a system remains an ideal but challenging target [9].

Covalent organic frameworks (COFs), a class of crystalline porous materials constructed through covalent bonds of organic ligands, have emerged as a promising platform for electrocatalysts during CO_2_RR [16–18]. Catalytic sites embedded in building blocks could be readily introduced into the COF backbone by reasonably selecting metal complexes as the linkers. This bottom-up approach enables precise and rational control over the selectivity and activity of the catalysts. In addition, the metal centers and surroundings can be finely tuned to modulate their electronic structures and interactions with key reaction intermediates for optimal electrocatalytic performances compared with inorganic metal catalysts [19–21]. Furthermore, the intrinsic ultrahigh surface area and porosity of COFs allow optimal exposure of active sites for high selectivity and activity in electrocatalytic CO_2_RR. A few previous studies have demonstrated that different metal nodes introduced into the COFs as active sites influence the electronic properties of COFs, subsequently, the overall performance of COFs in electrochemical CO_2_RR [22]. In fact, the chemistry of linkages also indeed plays a crucial role in determining the properties of COFs by modulating the extent of π-conjugation, planarity, conductivity, redox property, and stability of the reticular framework [23], offering a key factor to alter the electronic structure on single-metal sites based on modification beyond the first coordination sphere [24]. In addition, since linkages are located close to catalytic metal centers, varying the chemistry of linkages is supposed to be an effective alternative to tune the electronic characteristics and the resulting reactivity of metal active sites compared with the direct utilization of different metal sites [25, 26]. As a result, attempts have been made to construct COFs with different linkages [27–30] or through post-modification procedures [31]. Nevertheless, it is worth noting that few studies have explored the impact of different linkages on the electrical property and electrocatalytic performance of COFs within a system where other factors, including the identity of metal active sites, the type of conjugation, and functional groups, are well controlled.

Nickel phthalocyanine (NiPc) has a well-defined, open metal site, and a clear metal–N_4_ coordination structure, offering advantages of low cost, feasible accessibility, and good thermal and chemical stabilities. Among various MPcs with appropriate ligand π electrons, NiPc has received particular attention since Ni center has balanced binding energies for key intermediates, like *COOH and *CO, so that *CO is readily produced and released from the catalyst surface [31]. Considering this, in this study, we select the NiPc unit as the electrocatalytic module to incorporate it into COFs and showed that a single-point structural alteration in the linkage within a NiPc-based series effectively modulates the catalytic performance of the COFs in electrochemical CO_2_RR. This NiPc-based COF series with three members, named NiPc-TAB, NiPc-THB, and NiPc-TTB, which possess the same NiPc unit but different linkages, including piperazine, dioxin, and dithiine, have been constructed by nucleophilic aromatic substitution (S_N_Ar) reaction between 2,3,9,10,16,17,23,24-octafluorophthalocyanine nickel(II) (NiPc-F) and 1,2,4,5-tetraaminebenzene (TAB), 1,2,4,5-tetrahydroxybenzene (THB), and 1,2,4,5-tetrathiolbenzene (TTB), respectively. In our NiPc-based COFs, the linkages with different electron-donating heteroatoms bridged the outer rim of NiPc units, resulting in distinct electronic properties of Ni active sites. Among these COFs, the dioxin-linked COF NiPc-THB shows the best activity of CO_2_RR with a current density of CO (*j*_CO_) =  − 27.99 mA cm^−2^ at − 1.0 V (versus reversible hydrogen electrode, RHE). In addition, NiPc-TAB with the piperazine linkage demonstrates an excellent selectivity of Faradaic efficiency for CO (FE_CO_) = 90.7% at a low overpotential of 0.39 V. Moreover, NiPc-TAB achieves a high cathodic energy efficiency of > 65% toward CO products from − 0.5 to − 0.8 V (versus RHE), surpassing that of the other two NiPc COFs (20%–60%). Mechanistic studies by density functional theory (DFT) computation demonstrate that tailorable atomic-level tuning within a large framework could effectively alter the electronic states of active sites in NiPc-based COFs and engender distinct adsorption and desorption energy with crucial intermediates, thus acquiring modulated activity and selectivity in CO_2_RR.

## Experimental Section

### Synthesis of NiPc-Based COFs

**Fig. 1:**
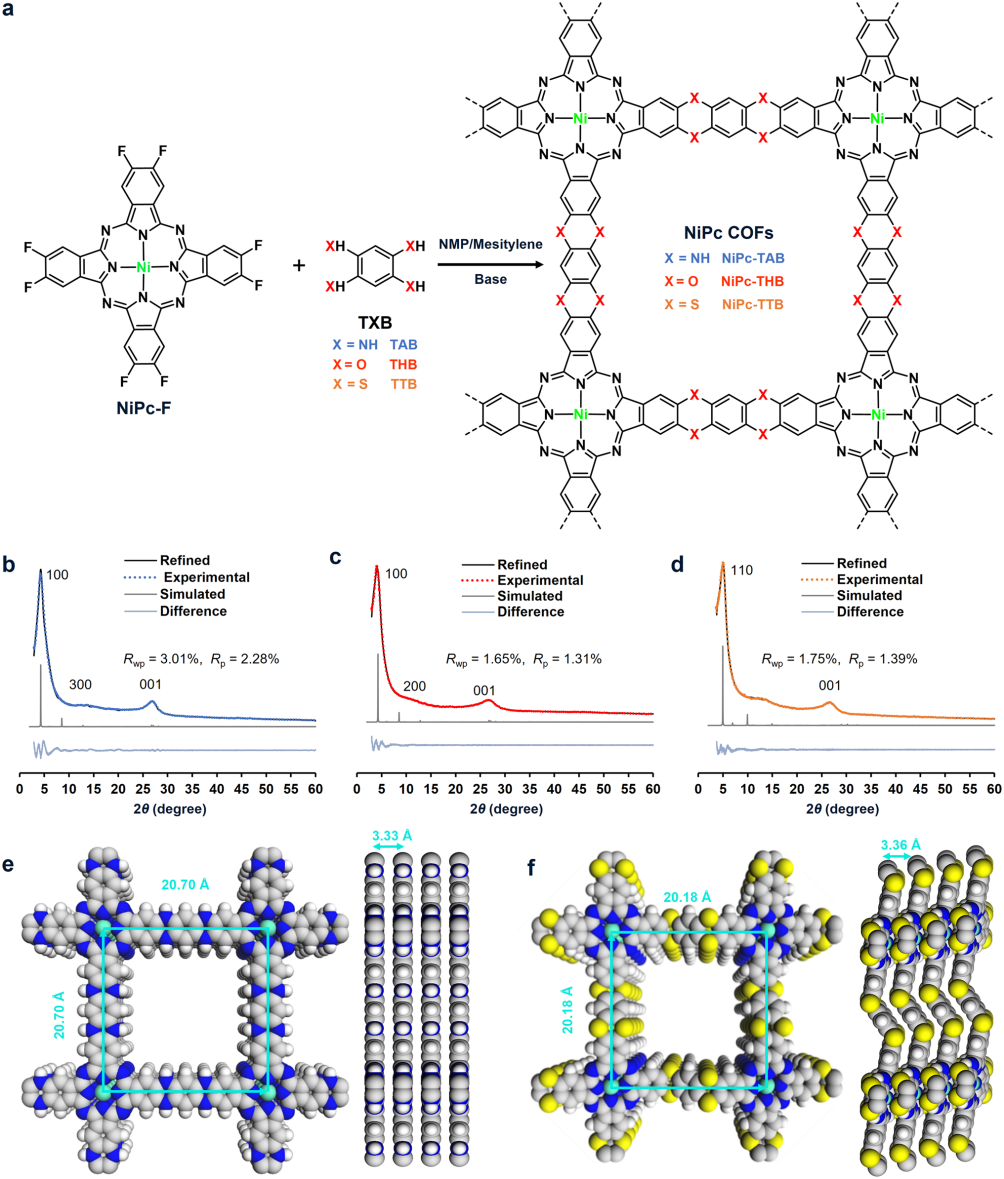
Synthetic scheme, structures, and characterization of the three NiPc-based COFs with different linkages. **a** Synthetic scheme of NiPc-TAB, NiPc-THB, and NiPc-TTB. Experimental profile (dot lines), refined profile (black solid lines), the difference between the experimental and refined PXRD (grayish blue solid lines), and simulation patterns (gray solid lines) of **b** NiPc-TAB, **c** NiPc-THB, and **d** NiPc-TTB. The simulated AA stacking structures of **e** NiPc-TAB and **f** NiPc-TTB (C, gray; N, blue; S, yellow; Ni, cyan; H, white)

The synthetic details of the building blocks, including NiPc-F, TAB, THB, and TTB, were described in the supporting information (Figs. S1-S4, Schemes S1–S3, and Table S1). The synthesis of the COFs involved the S_N_Ar reaction between NiPc-F and the corresponding tetrasubstituted benzene (Fig. 1a). Specifically, the linkers, TAB, THB, and TTB (0.08 mmol for each) were, respectively, added to Pyrex tubes charged with NiPc-F (0.04 mmol) in a mixed solvent of *N*-methylpyrrolidone (NMP) and mesitylene (1.5 mL: 1.5 mL) in the presence of the corresponding bases, which were 1,8-diazabicyclo[5.4.0]undec-7-ene for NiPc-TAB, 4-dimethylaminopyridine for NiPc-THB, and triethylamine for NiPc-TTB. The tubes were then frozen in a liquid N_2_ bath and sealed under vacuum. After reacting at 180 °C for three days, the resulting precipitates were collected by centrifugation and then thoroughly rinsed with organic solvents in a Soxhlet extractor for 1 week. After drying under vacuum for 24 h, NiPc-TAB, NiPc-THB, and NiPc-TTB were obtained as dark-blue powders in yields of 33%, 75%, and 45%, respectively.

### Preparation of the Working Electrode

COF inks were prepared by dispersing 4 mg of corresponding COF and 4 mg of carbon black (Macklin, C915132, cabot vulcan xc-72R, 10–20 nm) in a mixture of 40 μL of a Nafion 117 solution (D520 Dupont, 5 wt%) and 400 μL EtOH with the assistance of sonication for 30 min to form a homogeneous COF ink. Then, 200 μL ink was pipetted onto carbon fiber paper (CFP) (HCP020P, Toray) with a mass loading of 1.5–2 mg cm^−2^, which was further dried at room temperature for use. For examining the influence of different catalyst loading, the mass loading of NiPc-TAB has been controlled at 0.2–2.0 mg cm^−2^ by dropcasting different volumes of COF suspension.

## Results and Discussion

### Structural Characterizations of NiPc COFs

**Fig. 2:**
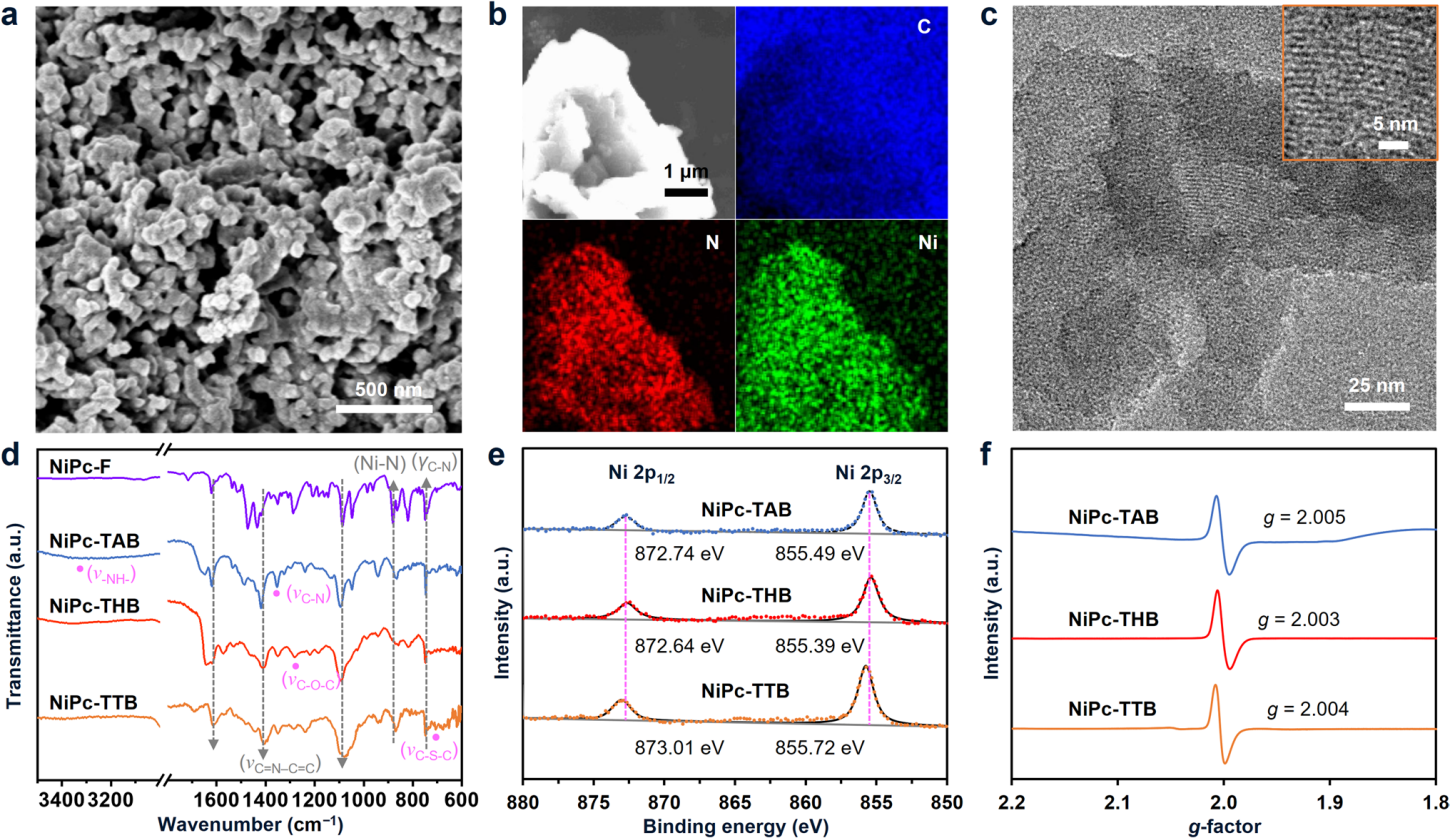
Morphology and electronic structure characterizations of the NiPc-based COFs. **a** SEM images of NiPc-TAB, **b** Typical SEM image and the corresponding elemental mappings of the C, N, and Ni elements, respectively, for NiPc-TAB. **c** HR-TEM images of NiPc-TTB. **d** ATR-IR spectra, **e** high-resolution XPS spectra of Ni 2*p*, and **f** EPR spectra of the COFs

The synthetic route of the three COFs is shown in Fig. 1a. Even though S_N_Ar reaction is not recognized as reversible as many of the well-known polycondensation reactions for imine, imide, or oxazole linkage formation for conventional COF synthesis [32], powder X-ray diffraction (PXRD) patterns verified the successful construction and reasonable crystallinity of the three COFs made by S_N_Ar reaction. The PXRD patterns of NiPc-TAB and NiPc-THB both showed strong diffraction peaks at around 4.3° and 26.8°, corresponding to (100) and (001) facets, respectively (Fig. 1b, c). The experimental patterns agreed with the simulated models based on AA stacked structures in the *P4/MMM* space groups with cell parameters of *a* = *b* = 20.70 Å, *c* = 3.33 Å,* α* = *β* = *γ* = 90° for NiPc-TAB and NiPc-THB (Figs. 1e and S5, S6). The PXRD pattern of NiPc-TTB showed two peaks at 4.9° and 26.6°, corresponding to (110) and (001) facets, respectively (Fig. 1d). The experimental PXRD pattern showed the best match with the simulation using a model with a nonplanar and wave-like configuration of dithiine units [32, 33] rather than a three-dimensional interpretated structure (Figs. S7 and S8), giving lattice parameters of *a* = *b* = 25.50 Å, *c* = 3.31 Å,* α* = *γ* = 90°, and *β* = 76.72° in *C2/M* space group for NiPc-TTB (Fig. 1f). Due to the presence of a dihedral angle of ca. 101.47° between the C–S bonds in the dithiine linkages, the side length of square formed by the Ni centers in NiPc-TTB is smaller than those in NiPc-TAB and NiPc-THB (20.18 versus 20.70 Å). Scanning electron microscopy (SEM) images showed that all the three COFs exhibited nanometer-sized irregular particles which were further aggregated into submicrometer-sized chunks (Figs. 2a and S9, S10). Energy-dispersive X-ray spectroscopy mappings of the three COFs revealed a homogeneous distribution of elements C, N, O, and S, as well as the metal element, verified the existence of desired composition of the COFs (Figs. 2b and S9, S10). High-resolution transmission electron microscopy (HR-TEM) provided direct visualization of crystal lattices with a side length of ∼1.9 nm for NiPc-TAB and NiPc-THB and ∼1.7 nm for NiPc-TTB (Figs. 2c and S11, S12), matching their (100) and (110) plane distances calculated from PXRD, respectively.

The formation of NiPc-based COFs was further confirmed by attenuated total reflectance infrared spectroscopy (ATR-IR) and elemental analysis. Besides the characteristic peaks from Ni–N, C–N, and C=N–C=C vibrations of the phthalocyanine backbones [34, 35] (see detailed assignments in Table S3), peaks from the dioxin, piperazine, and dithiine bridges were also observed, with the bands at 1336 and 3327 cm^–1^ ascribed to the stretching vibration of C–N bond [36] and N–H bond [36–38] in piperazine linkage, the band at 1281 cm^−1^ corresponding to the vibration of C–O bonds [33] in dioxin linkage, and the band at 704 cm^–1^ attributed to the stretching vibration of C–S bonds in dithiine linkages [32, 33] in NiPc-TAB, NiPc-THB, and NiPc-TTB, respectively (Fig. 2d). The Ni contents of NiPc-TAB, NiPc-THB, and NiPc-TTB determined by inductively coupled plasma atomic emission spectrometer (ICP-AES) were 5.4, 5.0, and 5.8 wt%, respectively, close to the theoretical values (Table S1). The thermogravimetric analysis of NiPc COFs showed less than 20% loss of the weight at the temperature of 400 °C (Fig. S13), suggesting the good thermostability of three COFs. N_2_ gas sorption isotherms at 77 K were performed to assess the porosity and specific surface area of the COFs. The Brunauer–Emmett–Teller (BET) surface areas for NiPc-TAB, NiPc-THB, and NiPc-TTB were 20, 45, and 138 m^2^ g^−1^, respectively (Fig. S14). The calculated pore sizes of these three COFs were about 1.5–2 nm, generally consistent with the theoretical values.

### Electronic Properties of NiPc COFs

The X-ray photoelectron spectroscopy (XPS) survey spectra of these three COFs disclosed the peaks from Ni, C, N, O, and S elements expected to present in the three COFs (Fig. S15). In N 1*s* spectrum of NiPc-TAB, the peak at 400.78 eV ascribed to C–N–H bond [36] was observed (Fig. S16b), confirming the formation of the piperazine linkage in NiPc-TAB. The peaks at 531.47 and 533.38 eV in the O 1*s* spectrum of NiPc-THB (Fig. S17c) were consistent with C–O–C bond [33] in dioxin linkage. Furthermore, two peaks observed at 163.92 and 165.11 eV in the S 2*p* spectrum of NiPc-TTB (Fig. S18c) corresponded to C–S–C bond [33] in dithiine linkages. Ni 2*p* XPS spectra demonstrated that the binding energy of Ni 2*p*_1/2_ and Ni 2*p*_3/2_ slightly shifted to higher values in a sequence of dioxin-, piperazine-, and dithiine-linked COFs (Fig. 2e). Compared with NiPc-THB, the higher binding energy of Ni 2*p* in NiPc-TAB might be due to the lower electronegativity N atoms in the piperazine linkage than O atoms in the dioxin linkage of NiPc-THB [39], while the higher binding energy in NiPc-TTB was likely caused by the electron-accepting effect of dithiine [31, 33, 37]. Consistent with the electronic state of Ni sites, N 1s XPS spectra of N atoms closest to Ni in NiPc-TAB and NiPc-TTB demonstrated binding energies at 399.60 and 399.54 eV, lower than that of NiPc-THB at 400.99 eV. These results suggested that varying the type of linkage influenced the electronic state of Ni sites in these COFs [25, 26], which was supposed to result in different abilities for CO_2_ activation.

Electron paramagnetic resonance (EPR) spectra (Fig. 2f) of NiPc-TAB, NiPc-THB, and NiPc-TTB exhibited strong and narrow EPR signals at *g* values of 2.005, 2.003, and 2.004, respectively, characteristic of organic radicals centered at the NiPc unit [40]. Solid-state ultraviolet–visible–near infrared (UV–Vis-NIR) spectra were conducted to investigate the absorption bands and optical band gaps of the COFs (Fig. S19). The adsorption of three NiPc-based COFs all exhibited prominent absorption at UV–Vis ranges which further extended to NIR range. NiPc-TAB, NiPc-THB, and NiPc-TTB showed significant absorption peaks in the visible region near 597, 612, and 576 nm, respectively, corresponding to the ligand-to-metal charge transfer in NiPc-based COFs [41]. The estimated band gaps assuming an indirect band gap by Tauc plots for NiPc-TAB, NiPc-THB, and NiPc-TTB were found to be 0.50, 0.72, and 0.52 eV, respectively (Fig. S20). These low band gap values suggested favorable charge transfer in these COFs, beneficial for their applications as catalytic electrode materials in electrochemical CO_2_RR. Two-point probe measurement gave conductivity values of 2.8 × 10^−5^, 3.3 × 10^−5^, and 3.4 × 10^−5^ S cm^−1^ for NiPc-TAB, NiPc-THB, and NiPc-TTB (Fig. S21), respectively, implying comparable electron conducting capabilities of the three NiPc COFs.

### Electrocatalytic CO_2_RR Performance of the COFs

**Fig. 3:**
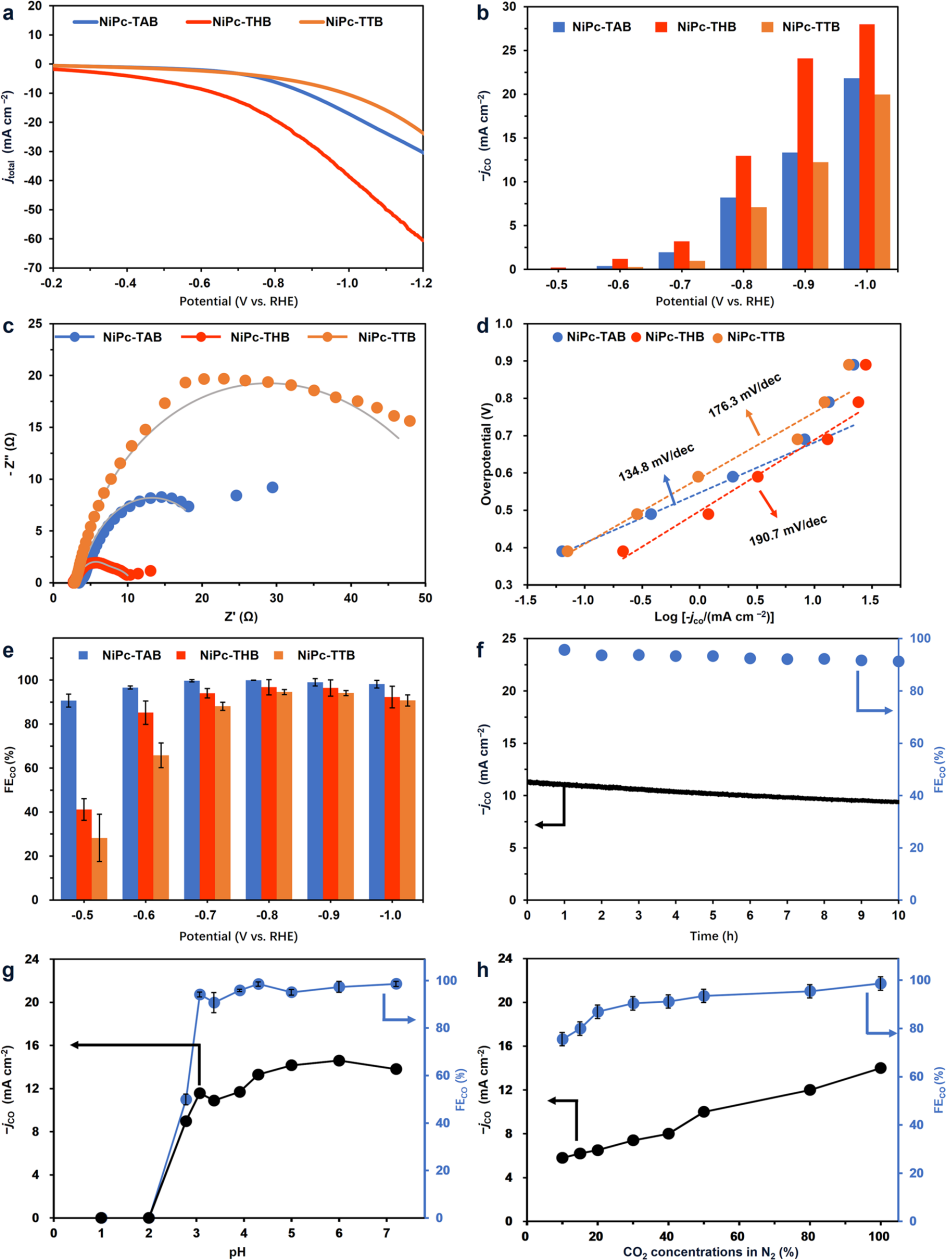
Electrochemical performance for CO_2_ reduction using the three NiPc-based COFs. **a** LSV curves of NiPc-based COFs in a CO_2_-saturated (pH = 6.8) 0.5 M KHCO_3_ solution. **b** Steady-state *j*_CO_ for the three COFs at different potentials. **c** Nyquist plots of NiPc-based COFs at a bias voltage of − 0.8 V (versus RHE). **d** Tafel plots for NiPc-based COFs. **e** FE_CO_ for the three COFs at different potentials. **f** Long-time electrolysis of NiPc-TAB at − 0.8 V (versus RHE). **g** FE_CO_ and *j*_CO_ of NiPc-TAB under different pH in an H cell at − 0.8 V (versus RHE). **h** FE_CO_ and *j*_CO_ of NiPc-TAB under different concentrations of CO_2_

The electrochemical CO_2_RR test was performed in an H-type cell separated by a proton exchange membrane with a 0.5 M aqueous solution of KHCO_3_ (pH = 7.2) as the electrolyte. The working electrode was prepared by depositing the homogeneous COF inks, which were made by dispersing the corresponding COF, carbon black, and a Nafion solution (5 wt%) in EtOH, and then drop-casted onto the carbon fiber electrodes (see also Sect. 2.2). Pt plate and Ag/AgCl electrode were used as the counter electrode and the reference electrode. The linear sweep voltammetry (LSV) of the COFs was first conducted from − 0.2 to − 1.2 V (versus RHE) at a scan rate of 10 mV s^–1^ under N_2_ and CO_2_ atmospheres, respectively (Figs. 3a and S22). For all of these three COFs, the current densities were significantly increased with the increased overpotential under CO_2_ atmosphere, indicating their potential CO_2_ catalytic activities [9, 17]. Moreover, the total current density (*j*_total_) in the LSV curves for NiPc-THB was much larger than that of NiPc-TAB and NiPc-TTB (Fig. 3a), indicating that dioxin-linked COF might have superior electrochemical CO_2_RR capability among the three COFs. The activity and selectivity of the COFs for CO_2_RR were then analyzed at the fixed potentials from − 0.5 to − 1.0 V (versus RHE). The gaseous products were quantified by gas chromatograph and the possible liquid products were analyzed by nuclear magnetic resonance (NMR). NMR spectra showed no presence of liquid product (Fig. S23) and CO was found as the exclusive product from the CO_2_RR with H_2_ as the byproduct from the HER.

The steady-state current densities increased with the applied voltage for the three COFs. At each potential, NiPc-THB gave the highest current density than the other two COFs. At − 1.0 V (versus RHE), a *j*_total_ of − 30.32 mA cm^–2^ was achieved in NiPc-THB with a partial current density for CO production (*j*_CO_) of − 27.99 mA cm^–2^ (Fig. 3b). This high *j*_CO_ demonstrated the excellent activity of NiPc-THB for electrochemical CO_2_RR, higher than those of some state-of-the-art reticular materials [17, 31, 37, 42–47], including COF-367-Co [17], MOF-1992 [44], CoPc-PDQ-COF [46], and *p*CoNiPc [22], the molecular MPc and MPy catalysts [8, 48–51], as well as MPc- and MPy-based composites [52–55] at similar conditions (Table S5). As for current densities for NiPc COFs in this work, compared with COFs with similar metal centers and ligands, the average steady-state total current density at the potential of − 0.8 V (versus RHE) reached − 8.2, − 13.4, and − 7.5 mA cm^−2^ of NiPc-TAB, NiPc-THB, and NiPc-TTB, respectively. These current densities achieved in this study were much higher than many reported COF examples and even comparable with that obtained by CoPc-PDQ-COF [46], a benchmark COF electrocatalyst at present at similar potentials (see Table S5). Moreover, plotting the *j*_CO_ on a logarithmic scale versus thermodynamic overpotential at low overpotential ranges gave Tafel slopes of 134.8, 190.7, and 176.3 mV dec^–1^ for NiPc-TAB, NiPc-THB, and NiPc-TTB (Fig. 3d), respectively, which are close to that of the one-electron reduction in the rate-limiting step for CO_2_-to-CO conversion on the electrode surface according to Volmer–Heyrovsky mechanism (118 mV dec^–1^). These Tafel slopes also located at the lower end of the value range for carbon catalysts [13], metal catalysts [19], and other MPy- and MPc-based COFs [29] in CO_2_RR (typically spanning 100–600 mV dec^–1^) (Table S5), indicating that the formation of CO catalyzed by these NiPc COFs was kinetically favorable [17], consistent with the observed trend of the activity of the COFs. Further, a lower CO formation kinetic barrier on NiPc-TAB was observed than on other two NiPc COFs. Electrochemical impedance spectra (EIS) in Fig. 3c (see also Fig. S24 and Table S4) showed that NiPc-THB had the lowest interfacial charge-transfer resistance (*R*_ct_) compared with other two NiPc-based COFs, indicating the fastest electron transfer from the catalyst surfaces to the reaction species. The result was consistent with the tendency of observed current density (Fig. 3c), implying that the charge-transfer resistance may be crucial to the total activity in the CO_2_RR test. Furthermore, double-layer capacitance values of NiPc-TAB, NiPc-THB, and NiPc-TTB were calculated to be 24.8, 82.9, and 15.1 mF cm^–2^, respectively, suggesting the presence of more catalytic sites in NiPc-THB than the other two COFs (Fig. S25). These values related to electrochemically active surface area combining with the analysis of the EIS and the Tafel plot, suggested feasible charge transfer of NiPc COF series in mediating CO_2_RR.

The FE_CO_ reached over 88% for all the three NiPc-based COFs got − 0.7 V (versus RHE). When more negative potentials were applied, the FE_CO_ reached 95%–99%, demonstrating these NiPc-based COFs as a group of highly selective CO_2_-to-CO catalysts over a wide potential range. Notably, NiPc-TAB also reached high FE_CO_ values over 90% under a wide potential range from − 0.5 to − 1.0 V (versus RHE), suppressing some representative MPc- and MPy-based COFs at similar conditions, including CoPc-PDQ-COF [46], NiPc-TFPN COF [27], CuPcF_8_-CoNPc-COF [56], CoPc-2H_2_Por COF [57], and NiPc-NH-TFPN-NH_2_ [31]. Among the three COFs, NiPc-TAB showed a high FE_CO_ of 90.7% at − 0.5 V (versus RHE), corresponding to a quite low overpotential of 0.39 V, illustrating the catalyst's ability to facilitate CO production from CO_2_ with minimal energy input [9]. Furthermore, as shown in Fig. S27, NiPc-TAB achieved a high cathodic energy efficiency of > 65% toward CO at the potential range of − 0.5 to − 0.8 V (versus RHE), much larger than those of NiPc-THB and NiPc-TTB at the same condition, demonstrating its advantage in energy consumption or economic viability for practical application. Under a fixed potential of − 0.8 V (versus RHE), the current density and FE_CO_ of NiPc-TAB retained over 91% for 10 h with a slight current density decrease (Fig. 3f), exhibiting the reasonable stability of NiPc-TAB. The slight decline of current density might arise from multiple reasons, including the shedding of COF catalysts, the leaching out of traces Ni [22], the tiny decrease in CO_2_ concentration, the local pH variation, and the partial Ni site poisoned by generated CO.

PXRD patterns of the COF electrocatalysts before and after electrocatalysis showed no obvious difference regarding peak positions despite the slightly reduced peak intensity (Fig. S28). Despite the slightly weakened relative peak intensity in the PXRD patterns of COFs, possibly due to the presence of a large amount of carbon black added during the electrode preparation process, the unaltered positions of PXRD peaks combined with the unchanged morphology and composition evidenced by SEM (Figs. S29–S31), TEM (Figs. S32–S34), and XPS (Figs. S35–S37) verified the structural integration and crystallinity of the COFs largely remained after electrochemical test. Further, Ni 2*p* XPS spectra showed that the + 2 valance of Ni sites in NiPc-based COFs was exclusively still reserved after test.

Given the promising CO_2_RR performance using conventional conditions, the electrocatalytic CO_2_RR of NiPc-TAB was also conducted by using different loadings and under different pH and concentrations of CO_2_ (Fig. 3g, h) to examine the ability of this catalyst under harsh and practical conditions. It was found that even at the low mass loading of 0.2 mg cm^–2^, NiPc-TAB exhibited a FE_CO_ of 80% and a *j*_CO_ of − 4.0 mA cm^–2^ (Fig. S26). NiPc-TAB exhibited an FE_CO_ > 90% at a wide pH range of 3.07–7.20 (Fig. 3g). In addition, a FE_CO_ of 76% and a total current density of 5.8 mA cm^–2^ can be reached under a low CO_2_ concentration of 10% (Fig. 3h). With the increase in CO_2_ concentration, both FE_CO_ and current density steadily increased. The above results showed that NiPc-TAB indeed exhibited excellent overall electrocatalytic performance with balanced activity, selectivity, and energy efficiency, including *j*_CO_ (− 8.20 mA cm^–2^), FE_CO_ (~ 100%), EE_1/2_ (60%–70%), and stability, which positioned it among one of the most competitive candidates, especially among those reported COFs with similar metal centers and ligands [17, 31, 37, 43, 45–48] and MPc- and MPy-based materials [8, 26, 48–51, 58] under the similar conditions (see Table S5 for a detailed comparison).

### Mechanism Study

The above electrochemical tests showed that the NiPc-based COFs varying in heteroatoms exhibited linkage-dependent CO_2_RR performance. Among them, NiPc-THB demonstrated the best activity of CO_2_RR performance while piperazine-linked COF stood out for its excellent selectivity and moderately good activity. To investigate the catalytic mechanism and the underlying structure-performance relationship, a series of experiments and DFT calculations were performed.

We firstly tested their CO_2_ adsorption isotherms of the three COFs at 298 K. The CO_2_ uptake is paramount to CO_2_ conversion because CO_2_ adsorption can increase the local concentration of CO_2_ around the catalytically active sites of materials even at low CO_2_ concentrations in the bulk electrolyte. The preabsorbed CO_2_ may potentially be activated by the functional group existing in the materials, further improving catalytic efficiencies of COFs [36]. As shown in Fig. S38, the CO_2_ adsorption capacity was 21.02, 33.54, and 28.67 cm^2^ g^–1^ for NiPc-TAB, NiPc-THB, and NiPc-TTB at 1 bar, respectively. The largest CO_2_ adsorption of NiPc-THB might contribute to its good activity in the CO_2_RR through CO_2_ preconcentration [10].

We then adopted DFT calculations to analyze the band structure and density of states (DOS) of NiPc-based COFs. NiPc-TAB exhibited significant band dispersion of approximately 1.0 eV near the fermi level, leading to a zero-band gap. NiPc-THB displayed a very small indirect band gap of 0.048 eV in the Brillouin zone along the out-of-plane direction. In contrast, a much larger band gap of 0.94 eV was observed for NiPc-TTB (Figs. S39–S41), which might be ascribed to the wrinkled layers due to the nonplanar dithiine linkage [33]. The much smaller band gaps of the piperazine-linked COF NiPc-TAB and the dioxin-linked COF NiPc-THB than NiPc-TTB likely improve the electron transfer in the 2D conjugated plane and accelerate the CO_2_RR.

**Fig. 4:**
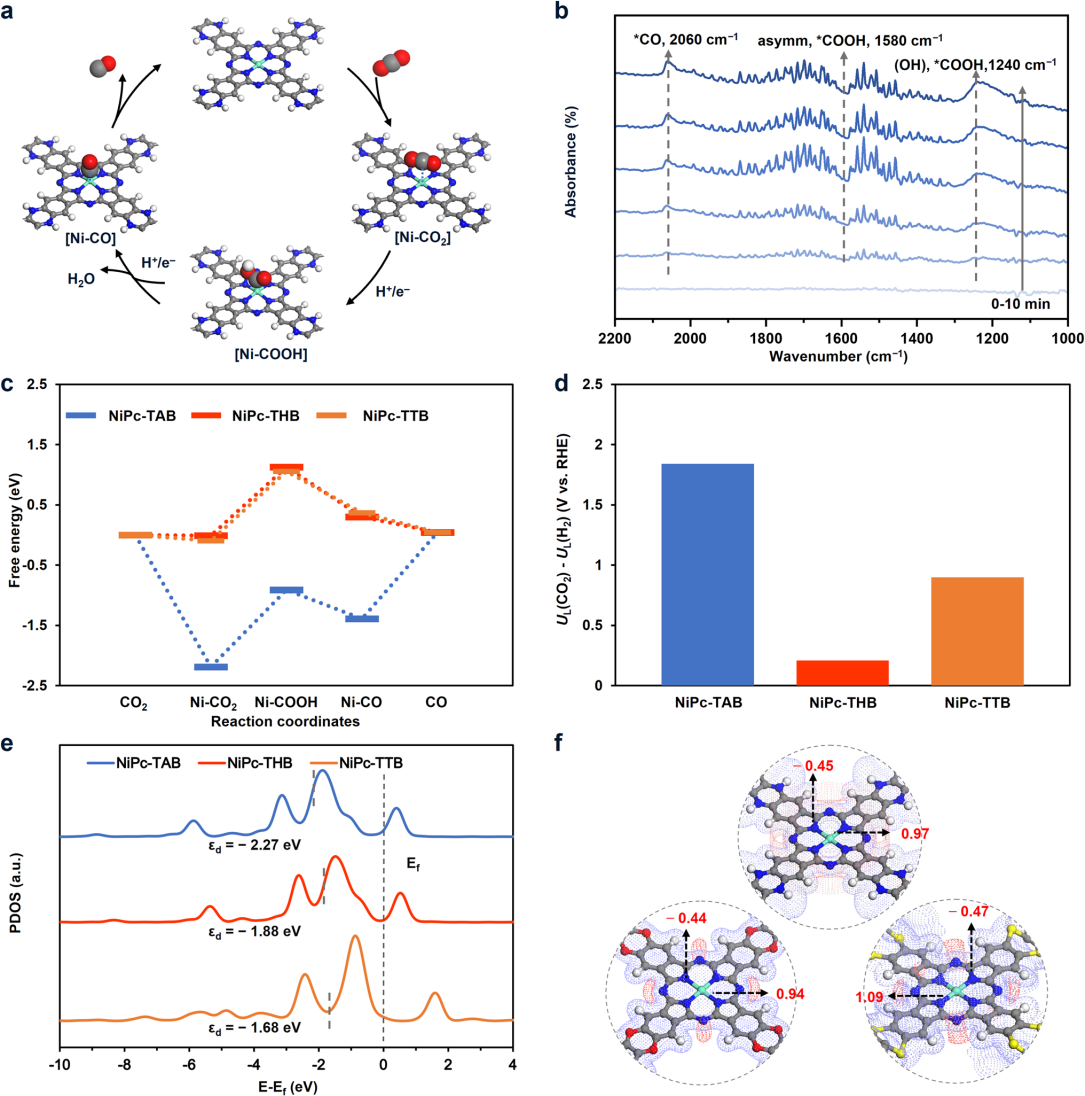
Mechanism study of NiPc-based COFs for CO_2_RR. **a** Proposed reaction pathway of the reduction of CO_2_ to CO. **b** *In-situ* FTIR spectra of NiPc-TAB collected at − 0.80 V (versus RHE) under CO_2_-saturated 0.5 M KHCO_3_ electrolyte. **c** Free energy diagrams of CO_2_RR, **d** the values of *U*_L_ (CO_2_)—*U*_L_ (H_2_), and **e** the projected d-band states of Ni atoms for NiPc-based COFs catalysts. **f** Mulliken charge analysis of the metal sites and the surrounding N atoms electrostatic potential diagram for NiPc-based COFs (isovalue 0.04 eV)

The CO_2_RR has been recognized as a two-proton–electron (H^+^/e^–^) transfer process consisting of four steps (Fig. 4a), including CO_2_ adsorption, the formation of carboxyl intermediate [Ni-COOH] (Ni represents the active site of the catalyst) through the first H^+^/e^–^ transfer process, the formation of CO adduct [Ni-CO] and water through the second H^+^/e^–^ transfer process, and CO desorption [2]. We observed the key intermediates involved in the above proposed mechanisms by *in-situ* infrared spectroscopy (Figs. 4b and S42), where the peaks at 1240 and 1580 cm^–1^ can be assigned to the OH and asymmetric stretching of the C=O stretching mode (*ν*_C = O_) of intermediate *COOH, while the peak at 2060 cm^–1^ corresponded to the adsorbed *CO [59].

DFT calculation was further adopted to gain additional insight into the catalytic process involved the possible intermediates mentioned. We adopted a single-layer model of the corresponding COFs to optimize the intermediates (Fig. S43) involved in the proposed pathway and to calculate their free energies (Tables S6–S8 for the calculation details). The calculated free energy (Δ*G*) diagram revealed that the rate-determining step (*E*_*a*_) of in the CO_2_-to-CO conversion for the NiPc-TAB was the desorption of CO while the rate-determining steps for NiPc-THB and NiPc-TTB in CO_2_RR were the formation of the [Ni–COOH] [45, 51]. The calculated energy barriers for the rate-determining steps were 1.43, 1.14, and 1.16 eV for NiPc-TAB, NiPc-THB, and NiPc-TTB, respectively (Fig. 4c). These *E*_a_ values were in good agreement with experimentally observed trend where NiPc-THB gave highest activity. The HER process was also simulated and combined with the CO_2_RR process to assess the selectivity in the CO_2_RR process (Fig. S44). The limiting potential difference between CO_2_RR and HER (*U*_L_ (CO_2_)—*U*_L_(H_2_), *U*_L_ =  − Δ*G*/e) has been calculated and used as a descriptor for evaluating CO selectivity [60]. As shown in Figs. 4c and S44, the *U*_L_ (CO_2_)—*U*_L_ (H_2_) values of NiPc-TAB, NiPc-THB, and NiPc-TTB were 1.84, 0.21, and 0.9 V, respectively, which matched well with the experimental results with NiPc-TAB possessing the best selectivity under a low overpotential in CO_2_RR (Fig. 3d). The discrepancy between the calculated results and experimental observations for NiPc-based COFs may be attributed to the state of the catalysts (e.g., the morphology, availability of the active sites, and the presence of defects) [61].

The projected density of states (PDOS) of 3*d* orbital for Ni sites in these NiPc-based COFs showed that the d-band centers of the Ni atom gradually shifted to the Fermi level when the linkages evolved from piperazine to dioxin and dithiine, giving d-band center value (*ε*_d_) of − 2.27, − 1.88, and − 1.68 eV, respectively, for NiPc-TAB, NiPc-THB, and NiPc-TTB (Fig. 4e), which means the different single-point linkages significantly alter the electron configuration within the Ni 3*d* orbitals [62, 63]. Based on the Sabatier principle, too strong or too weak adsorption is not conducive to improve the catalytic activity [64]. NiPc-THB with a moderate ε_d_ value might give appropriate adsorption between C-intermediates and Ni sites and promote the activity in CO_2_RR [65]. The calculated Mulliken charge of the Ni site in NiPc-THB was 0.94, lower than those in NiPc-TAB (0.97) and NiPc-TTB (1.09) (Fig. 4f), suggesting the alternative of linkages induced different electron density on the Ni sites, consistent with the electronic states of Ni 2*p*, the calculated Mulliken charge of the surrounding N atoms, and the binding energy of N 1*s* in N–Ni observed in XPS (Figs. 2e, 4f and S16–S18). The lower positive charge density and more negative electrostatic potential of the Ni sites in NiPc-THB might promote desorption of some intermediates compared with other two COFs to improve the electrocatalytic activity in CO_2_RR process [66, 67], while moderate charge density on the Ni sites in NiPc-TAB likely provides suitable adsorption and desorption for the intermediates to give good combination of activity and selectivity [19].

## Conclusions

In summary, a series of porous NiPc-based COFs with various linkages of dioxin, piperazine, and dithiine, respectively, were fabricated and investigated as a platform for CO_2_RR. Electrochemical measurements revealed that their electrocatalytic selectivity toward CO followed the order of NiPc-TAB > NiPc-THB > NiPc-TTB, with a difference of more than 60% between the piperazine and dithiine linkage under an overpotential of 0.39 V. Furthermore, NiPc-TAB demonstrates outstanding performance with a high FE_CO_ of 90.7% at an overpotential of 0.39 V, surpassing most COFs in CO_2_RR. This work not only expands the types of linkage-based MPc-COFs, but also confirms that the tuning of peripheral groups exhibits distinct electron density of active sites and alters the adsorption and desorption energy of the active site with key intermediates during CO_2_RR process, building upon the fundamental insight into the structure–property relationships of 2D framework materials, which should be beneficial to the design and preparation of high-performance and low-cost electrocatalysts for CO_2_RR. Our work highlights the significant potential of the utilization of porous conjugated COFs for the electrochemical conversion of CO_2_ to CO and paves the way for designing high-performance CO_2_ reduction catalysts through linkage engineering by strategically optimizing combinations of various structural factors.

## Supplementary Information

Below is the link to the electronic supplementary material.

Supplementary file1 (DOCX 11425 KB)
