# Supplementary material for: Single-Point Linkage Engineering in Conjugated Phthalocyanine-Based Covalent Organic Frameworks for Electrochemical CO2 Reduction
Source: Nanomicro Lett. 2025 May 9;17:252. doi: 10.1007/s40820-025-01754-9 (PMC12064513; doi:10.1007/s40820-025-01754-9)
Supplement: Supplementary file 1 — Supplementary file1 (DOCX 11425 KB) [file 40820_2025_1754_MOESM1_ESM.docx]

Supporting Information for

**Single-Point Linkage Engineering in Conjugated Phthalocyanine-Based Covalent Organic Frameworks for Electrochemical CO_2_ Reduction**

Wenchang Chen^1^, Yi Zhang^1^, Mingyu Yang^1^, Chao Yang^1^*, Zheng Meng^1^*

^1^Department of Chemistry, University of Science and Technology of China, Hefei, Anhui 230026, P.R. China

*Corresponding authors. E-mail: [zhengmeng@ustc.edu.cn](mailto:zhengmeng@ustc.edu.cn) (Zheng Meng); [chao_yang@ustc.edu.cn](mailto:chao_yang@ustc.edu.cn) (Chao Yang)

**S1 Materials**

All the starting materials were purchased from commercial source and used without further purification, including 1,2-dibromo-4,5-difluorobenzene, N,N-dimethylacetamide (DMAc), poly(methylhydrosiloxane) (PMHS), 1,1’-ferrocenediyl-bis(diphenylphosphine) (DPPF), tris(dibenzylideneacetone)dipalladium(0) (Pd_2_(dba)_3_), zinc cyanide (Zn(CN)_2_), magnesium sulfate (MgSO_4_), nickel(II) chloride (NiCl_2_), ethylene glycol, 2,5-dihydroxy-1,4-benzoquinone, hydrochloric acid (36 wt%), triethylamine (Et_3_N), 1,8-diazabicyclo[5.4.0]undec-7-ene (DBU), 4-dimethylaminopyridine (DMAP), tin, tetrahydrofuran (THF), sodium hydride, N,N-dimethylformamide (DMF), benzyl mercaptan, 1,2,4,5-tetrafluorobenzene, sodium, 1,2,4,5-tetraaminobenzene hydrochloride (TAB), dichloromethane (DCM), ethyl acetate (EtOAc), methanol, ethyl alcohol, and N-methylpyrrolidone (NMP), 1,3,5-trimethylbenzene (mesitylene), petroleum ether (PE), and acetone.

**S2 Instruments of materials characterization**

Nuclear magnetic resonance (NMR) experiments were recorded on a Bruker AVAVCE III HD400. Powder X-ray diffraction (PXRD) measurements were conducted with a Rigaku sixth generation multifunctional rotating-anode X-ray diffractometer. Scanning electron microscopy (SEM) images were obtained using a ZEISS GeminiSEM 450. Transmission electron microscopy (TEM) was carried out on a JEOL JEM-F200. Fourier transform infrared (FTIR) spectra were obtained using attenuated total reflectance infrared spectroscopy (ATR-IR) by Bruker Tensorll. Thermal gravimetric analysis (TGA) was performed using a NETZSCH TG 209F1 Libra. Elemental analysis, including C, H, N and S was performed on an Elementar Vario EL cube using a combustion method by automatic analyzers. The metal contents were analyzed by Thermo Fisher iCAP 7400 inductively coupled plasma atomic emission spectrometer (ICP-AES). X-ray photoelectron spectroscopy (XPS) experiments of each element in this NiPc COF series before and after electrocatalysis were recorded on Thermo Scientific ESCALAB 250Xi and Thermo NEXSA G2 X-ray Photoelectron Spectrometer. Electron paramagnetic resonance (EPR) spectra were obtained using a JEOL JES-FA 200 (150 K, 9.08 GHz, X-band) with a microwave power of 2 mW from 187.3 to 487.3 mT. Ultraviolet–visible–near infrared (UV–Vis–NIR) spectra between 200 and 2500 nm were acquired using a Shimadzu SolidSpec-3700DUV spectrophotometer. CO_2_ adsorption measurements were performed on a Micromeritics ASAP 2460BSD-PM2 physisorption analyzer at 298 K. Electrochemical measurements were conducted on a CH Instruments CHI 660E electrochemical analyzer. The electrochemical test was performed using a three-electrode setup in an H-shape glass cell separated by a SELEMION anion exchange membrane (Nafion115, Dupont). The gaseous products were determined and quantified by Shimadzu GC-2014 gas chromatography with a thermal conductivity detector and a flame ionization detector. *In-situ* FTIR in electrocatalytic CO_2_RR was conducted on Bruker Tensorll, using a customized sealed PTFE electrochemical cell with a gold electrode as the working electrode, Ag/AgCl electrode as reference electrode, platinum wire as the counter electrode, and Nafion 212 membrane as separate at room temperature.

**S3 Synthesis of NiPc-based COFs**

**S3.1 Synthesis of NiPc-F**

**Scheme S1** Synthesis of (2,3,9,10,16,17,23,24-octafluorophthalocyanine) nickel (II) (compound 3)

**S3.2 Synthesis of 4,5-difluoro-1,2-dicyanobenzene (compound 2)**

4,5-difluoro-1,2-dicyanobenzene (**2**) was synthesized from **1** according to literature methods [S1]. A 250 mL two-neck round-bottom flask was charged with 5.44 g (20.0 mmol) of **1** in DMAc (40 mL) and PMHS (400 mg, 2.00 mol%) at room temperature. The reaction mixture was heated to 100 ℃, followed by Pd_2_(dba)_3_ (400 mg, 2.00 mol%) and DPPF (300 mg, 2.70 mol%). Afterwards, Zn(CN)_2_ (3.52 g, 30.0 mmol) was added in 4 portions within 3 hours. The reaction mixture was heated for another 2 hours and then cooled down to room temperature, diluted with EtOAc and filtered. Filtrate was washed with H_2_O, dried with MgSO_4_, and concentrated in vacuum. The crude product was purified by column chromatography using PE/DCM (v/v=1/1) as an eluent to give a pale-yellow powder, which was dried in vacuum. (2.00 g, yield 61%). ^1^H NMR (400 MHz, CDCl_3_): *δ* = 7.68 (t, *J*_F-H_ = 8.0 Hz, 2H).

**Fig. S1** ^1^H NMR (400 M, 298 K, DMSO-*d*_6_) spectrum of **2**

**S3.3 Synthesis of (2,3,9,10,16,17,23,24-octafluorophthalocyanine) nickel (II) (compound 3)**

A mixture of **2** (1.64 g, 10.0 mmol), anhydrous NiCl_2_ (0.37 g, 2.86 mmol), and ethylene glycol (13 mL) was heated and stirred at 180 °C for 4 h under N_2_. After cooling to room temperature, the reaction mixture was treated with ethanol to precipitate the dark green product and then filtered. The precipitate was intensively washed with ethanol and acetone. Yield: 55%. Element analysis and ICP-AES for C_33_H_11_F_8_N_8_Ni (C, 54.3%; H, 1.5%; F, 20.8%; N, 15.4%; Ni, 8.0%), found: C, 53.1%; H, 1.9%; N, 15.5%; Ni, 12.5%.

**S3.4 Synthesis of 1,2,4,5-tetrahydroxybenzene (****THB, compound 5)**

**Scheme S2** Synthesis of THB (compound **5**)

THB (**5**) was synthesized from **4** according to literature methods [S2]. To a mixture of 32.2 g (230 mmol) of **4** in 700 mL of 36% hydrochloric acid, 32.8 g (276 mmol) of granular tin was slowly added. This mixture was slowly heated to reflux for 1 h, during which time the reaction mixture remained black. The solution was filtered through a coarse glass frit and allowed to cool slowly to room temperature and then cooled to 0 °C. The large colorless crystals of **5** were collected by filtration and dried in vacuum affording 24.4 g (75%) of a brown solid, which was recrystallized from THF into a white crystalline solid (20.3 g, 62%). ^1^H NMR (400 MHz, DMSO-*d*_6_): *δ* = 8.02 (br, 2H), 6.21 (s, 1H).

**Fig. S2** ^1^H NMR (400 M, 298 K, DMSO-*d*_6_) spectrum of **5**

**S3.5 Synthesis of** **1,2,4,5-tetrathiolbenzene (TTB, compound 8)**

**Scheme S3** Synthesis of TTB (compound **8)**

**S3.6 Synthesis of 1,2,4,5-tetrakis(benzylthio)benzene (compound 7)**

To a suspension of sodium hydride (3.52 g, 88.0 mmol) in dry DMF (20 ml), benzyl mercaptan (10.4 mL) was added under N_2_ while keeping the temperature between 10 and 15 °C. The mixture was brought to room temperature and **6** (1.1 mL, 10.0 mmol) was then added in small portions. The reaction mixture was left overnight under N_2_ while stirring. The product was collected by filtration and purified by column chromatography (silica gel, DCM/PE = 1/1 as an eluent). The last fraction was collected and then dried in vacuum. Finally, white product was collected for 2.77 g in 49% yield. ^1^H NMR (400 MHz, DMSO-*d*_6_): *δ* = 4.07 (s, 4H), 7.03 (s, 1H), 7.28 (m, 10H).

**Fig. S3** ^1^H NMR (400 M, 298 K, DMSO-*d*_6_) spectrum of **7**

**S3.7 Synthesis of TTB (compound 8)**

To 50 mL of anhydrous liquid ammonia at −78 °C, solid sodium (1.38 g, 60 mmol) was added in 10 portions (sodium stored in oil was rinsed by hexane and cut before use). A dark-blue color appeared as the sodium dissolved. Then, **7** (1.13 g, 2.0 mmol) was added under nitrogen atmosphere. The solution was stirred for 4 hours at −78 °C. Methanol (10 mL, degassed by bubbling nitrogen) was then added cautiously via a syringe to the flask until the blue color disappeared. The flask was warmed to room temperature over 2 hours. Subsequently, the yellow solid was then washed by DCM (degassed by bubbling nitrogen) and dispersed in 100 mL of deionized water (degassed by bubbling nitrogen). Then 10 mL of HCl (5%, degassed by N_2_) was then dropwise added into the above aqueous layer until yellow precipitate formed. The mixture was centrifuged at 8000 rpm/min for 10 min and the liquid was then decanted. The yellow solid was then washed by water (degassed by nitrogen, 3×100 mL) and acetone (degassed by nitrogen, 3×100 mL). The pale-yellow precipitate was then dried under vacuum for 24 hours. 371 mg (1.80 mmol) of yellow product was collected in 90% yield. The product was stored under vacuum. ^1^H NMR (400 MHz, CDCl_3_): *δ* = 3.99 (s, 2H), *δ* = 7.41 (s, 1H).

**Fig. S4** ^1^H NMR (400 M, 298 K, DMSO-*d*_6_) spectrum of **8**

**S3.8 Synthesis of** **NiPc-based COFs**

**Synthesis of NiPc-TAB.** NiPcF_8_ (28.56 mg, 0.04 mmol) and 1,2,4,5-tetraaminobenzene hydrochloride (TAB) (22.72 mg, 0.08 mmol) were added into the mixed solvent of 1.5 mL mesitylene and 1.5 mL NMP in a 16 mL Pyrex tube measuring o.d. × i.d. = 10 × 8 mm^2^. The mixture was sonicated for 5 min to form a homogeneous suspension. Then, 100 μL DBU was added into the mixture. After three freeze-pump-thaw cycles, the Pyrex tube was sealed and heated in an oven at 180 °C for 5 days. The dark-blue precipitate was collected by centrifugation and rinsed with acetone, DCM, and THF in a Soxhlet extractor for 1 week. The resulting COF was obtained as dark-blue powder in a yield of 33%.

**Synthesis of NiPc-THB.** NiPcF_8_ (28.56 mg, 0.04 mmol) and 1,2,4,5-tetrahydroxybenzene (THB) (11.37 mg, 0.08 mmol) were added into the mixed solvent of 1.5 mL mesitylene and 1.5 mL NMP in a 16 mL Pyrex tube measuring o.d. × i.d. = 10 × 8 mm^2^. The mixture was sonicated for 5 min to form a homogeneous suspension. Then 100 mg DMAP was added into the mixture. After three freeze-pump-thaw cycles, the Pyrex tube was sealed and heated in an oven at 180 °C for 3 days. The dark-blue precipitate was collected by centrifugation and rinsed with acetone, dichloromethane, and THF in a Soxhlet extractor for 1 week. The resulting COF was obtained as dark-blue powder in a yield of 75%.

**Synthesis of NiPc-TTB.** NiPcF_8_ (28.56 mg, 0.04 mmol) and 1,2,4,5-tetrathiolbenzene (TTB) (16.48 mg, 0.08 mmol) were added into the mixed solvent of 1.5 mL mesitylene and 1.5 mL NMP in a 16 mL Pyrex tube measuring o.d. × i.d. = 10 × 8 mm^2^. The mixture was sonicated for 5 min to form a homogeneous suspension. Then 100 μL Et_3_N was added into the mixture. After three freeze-pump-thaw cycles, the Pyrex tube was sealed and heated in an oven at 180 °C for 5 days. The dark-blue precipitate was collected by centrifugation and rinsed with acetone, DCM, and THF in a Soxhlet extractor for 1 week. The resulting COF was obtained as dark-blue powder in a yield of 45%.

**S4 Elemental analysis**

Elemental analyses, including C, H, S, N were performed by Elementar vario EL cube using a combustion method by automatic analyzers. The metal content was analyzed by Optima 7300 DV inductively coupled plasma atomic emission spectrometer (ICP-AES). After NiPc-TXB (X= O, NH, S) COFs were synthesized as described in section 1.4, it was further performed three solvent thermal activations using ethanol and water separately. Finally, these materials were dried in vacuum desiccator equipped with an oil pump for 24 h under 50 °C in preparation for elemental analysis. Elemental analyses, including C and H, were performed by Elementar vario EL cube using a combustion method by automatic analyzers.

**Table S1** Elemental analysis of NiPc-based COFs


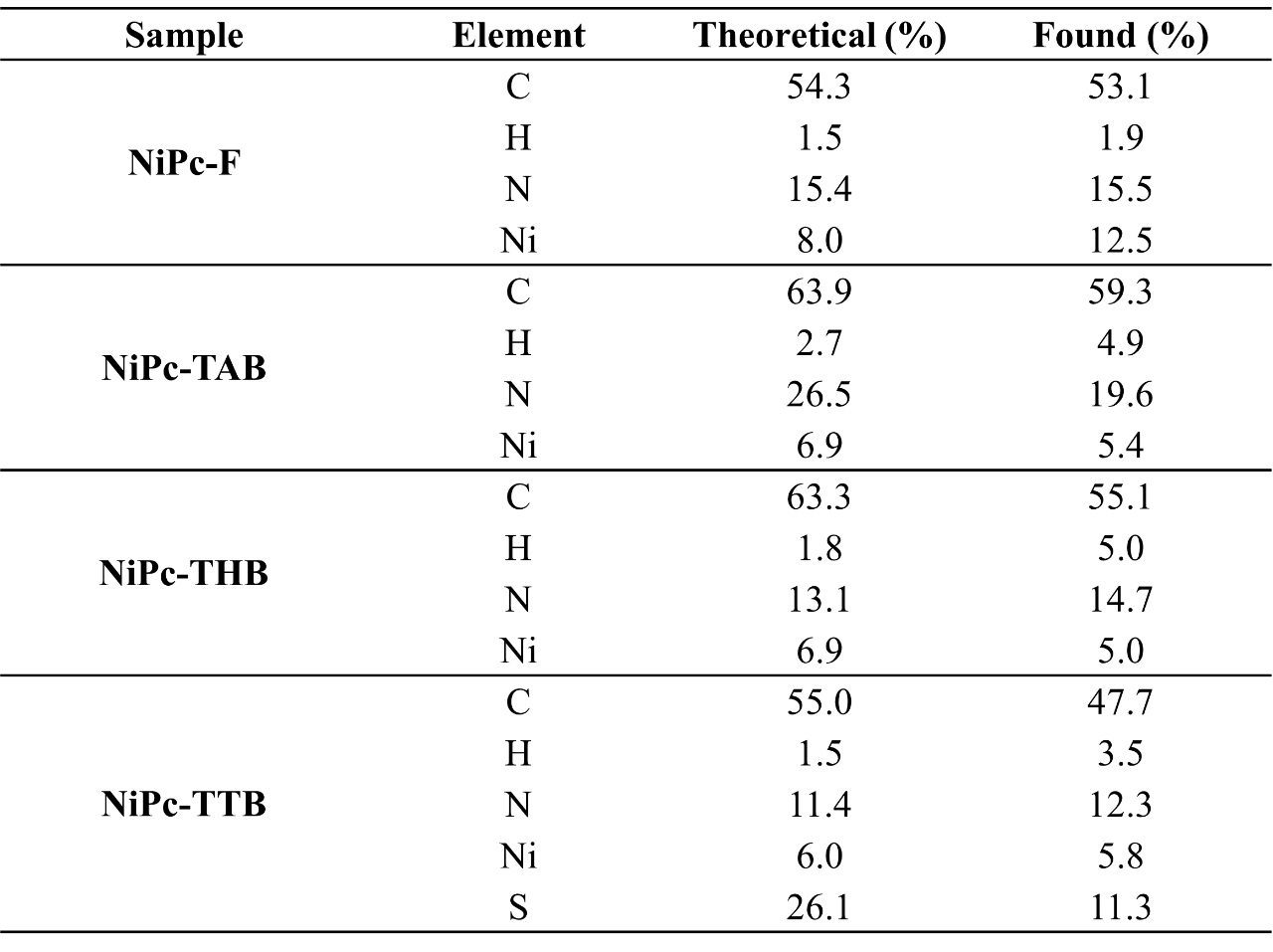


**S5 Structural analysis**


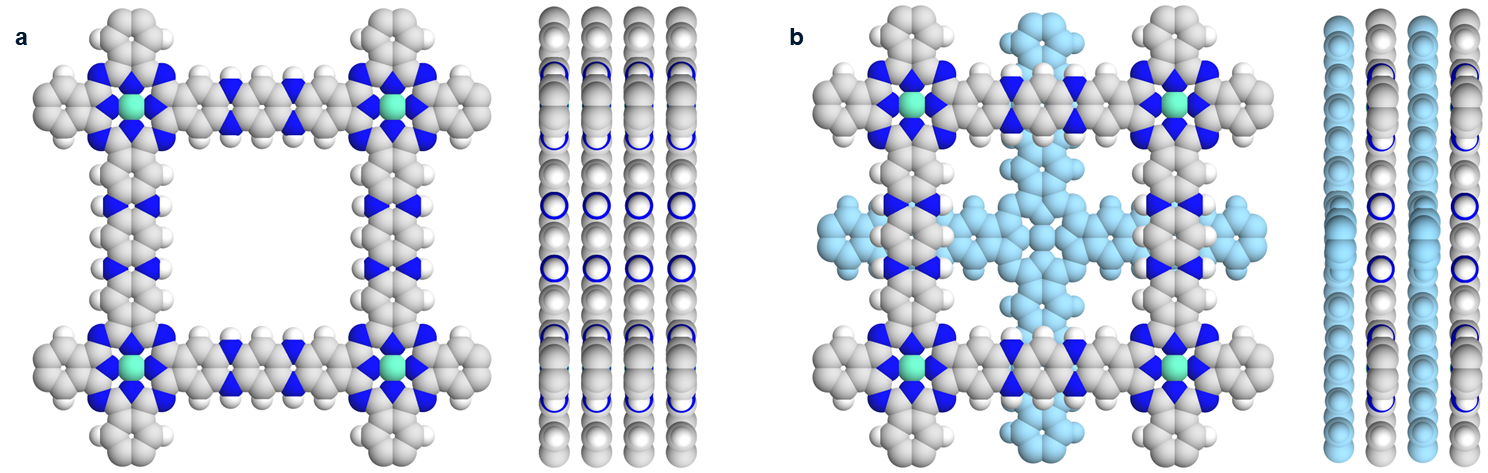


**Fig. S5** **a** AA stacking and **b** AB stacking views from z and x axes of modeled COF crystal structures with eclipsed and staggered packing mode for NiPc-TAB


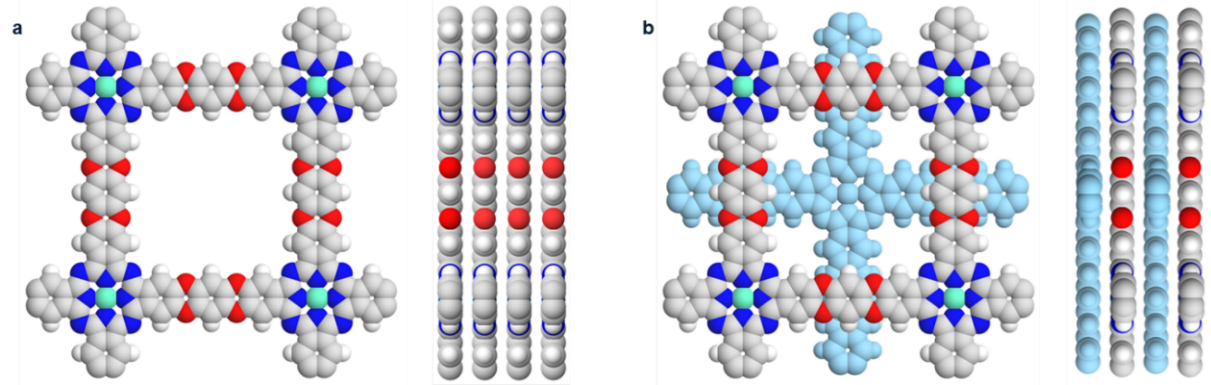


**Fig. S6 a** AA stacking and **b** AB stacking views from z and x axes of modeled crystal structures with eclipsed and staggered packing mode for NiPc-THB


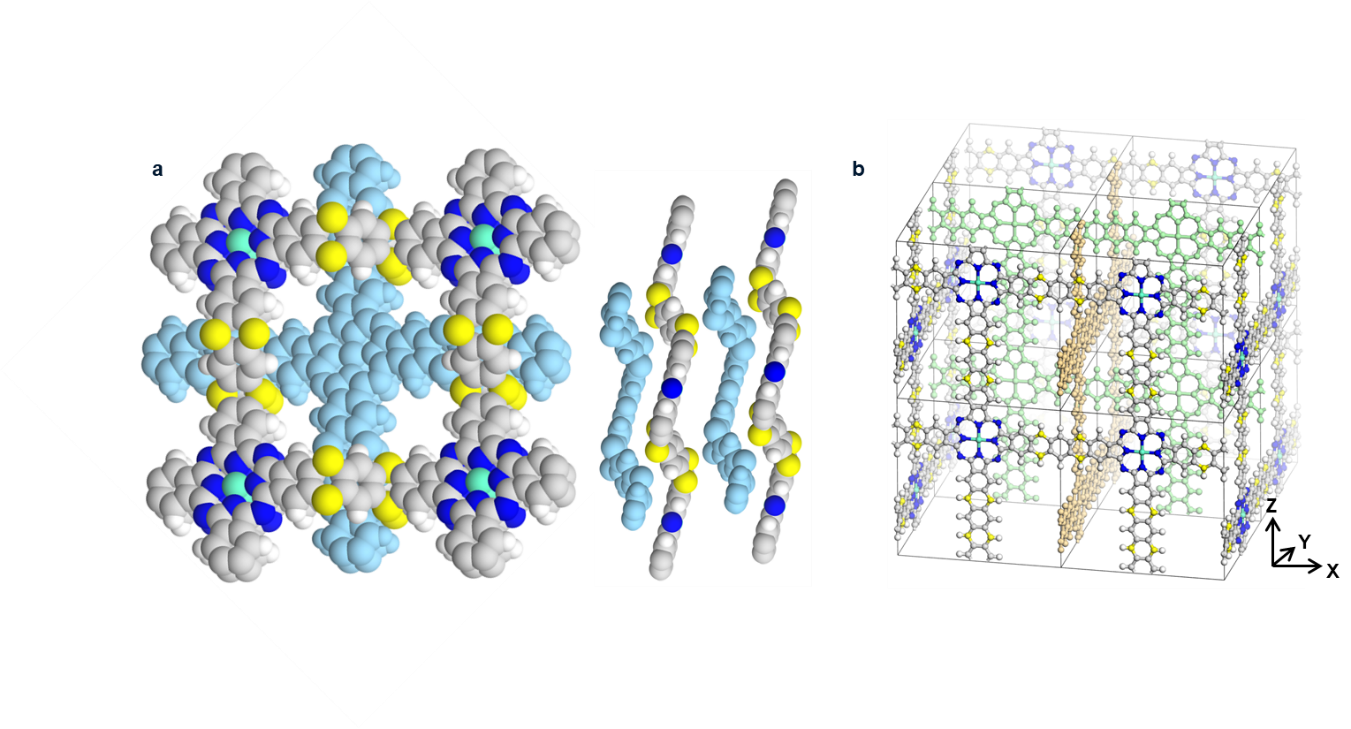


**Fig. S7** **a** AB stacking views from *z* and *x* axes of modeled COF crystal structures with staggered packing mode and **b** three-dimentional modeled COF crystal structures with structural interpretation viewing from *x*, *y*, and *z* axes for NiPc-TTB


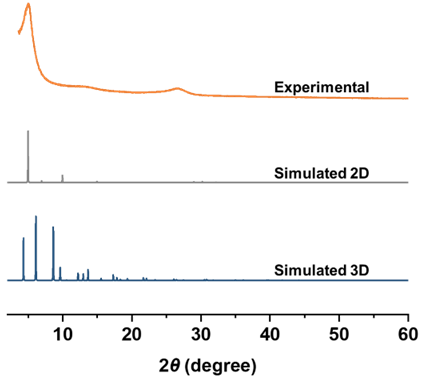


**Fig. S8** The comparison of simulated 2D AA stacking structure model and 3D interpretation mode patterns with the experimental profile of NiPc-TTB

The geometry optimization with cell parameters was performed using the CASTEP module in Materials Studio with Perdew−Burke−Ernzerhof (PBE) generalized gradient approximation (GGA). The convergence tolerance was set at 2×10^-5^ eV/atom and the max force was set to 0.05 eV A^-1^. Calculation of the simulated powder diffraction pattern was performed by Materials Studio Reflex Plus Module. The optimized crystal structure of NiPc-TAB and NiPc-THB are shown in Figs. 5 and 6 with lattice parameters a = b = 20.70 Å, c = 3.33 Å in *P4/MMM* space group. Lattice parameters of NiPc-TTB shows lattice parameters of a = 28.44, b = 28.64 Å, c = 3.81 Å, *α* = *γ* = 90°, *β* =76.72° in *C2/M* space. Modeling of the staggered structures was performed in a similar manner but with the space group *I4/mmm* for NiPc-TTB (Fig. S7).

**Table S2** The full width at half maxima (FWHM) and grain size calculated by Debye-Scherrer equation of three NiPc COFs

| **Parameter**  **COF** | **Full width at half maxima (FWHM)** | **Grain size (nm)** |
| --- | --- | --- |
| **NiPc-TAB** | 0.03301 | 4.16 |
| **NiPc-THB** | 0.03118 | 4.40 |
| **NiPc-TTB** | 0.03936 | 3.49 |

**S6 SEM and TEM images**

**
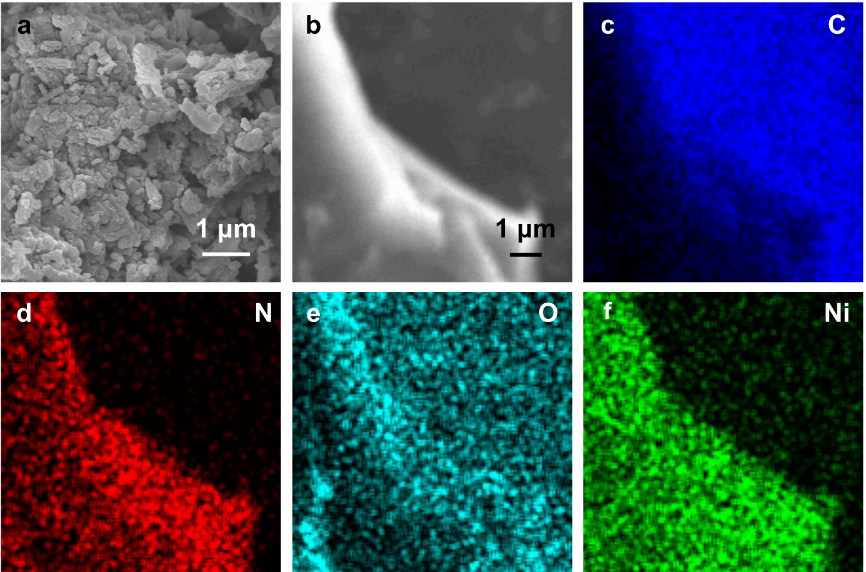
**

**Fig. S9** **a** SEM image of NiPc-THB. **b** SEM image and **c-f** corresponding elemental mappings of the C, N, O, and Ni elements for NiPc-THB, respectively

**
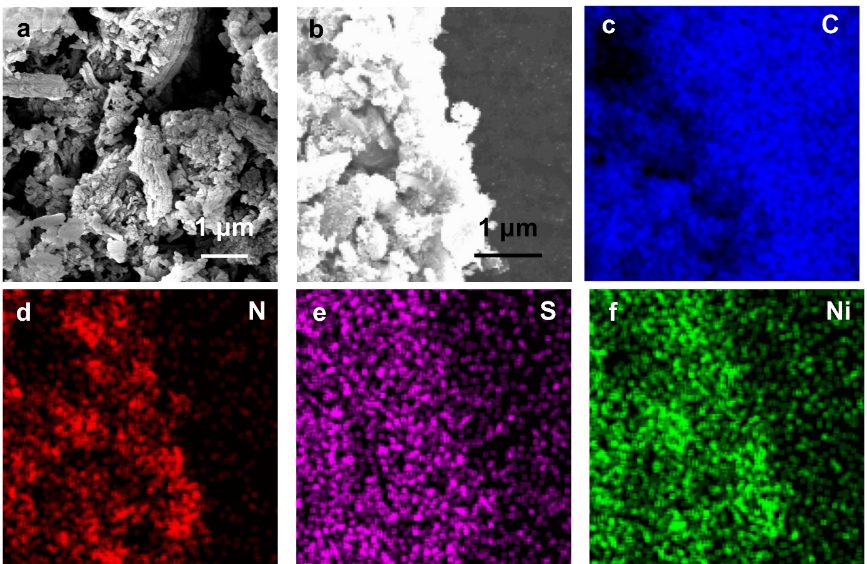
**

**Fig. S10** **a** SEM image of NiPc-TTB. **b** SEM image and **c-f** corresponding elemental mappings of the C, N, S, and Ni elements for NiPc-TTB, respectively


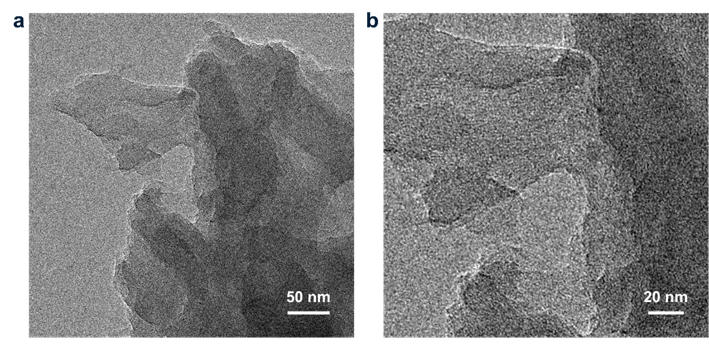


**Fig. S11** TEM images of NiPc-TAB at different magnifications

**
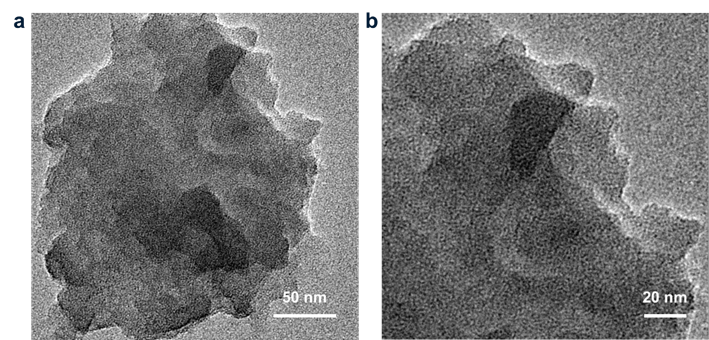
**

**Fig. S12** TEM images of NiPc-THB at different magnifications

**S7 ATR-IR spectra**

Fourier transform infrared spectra were obtained using an attenuated total reflectance infrared spectroscopy (ATR-IR) by Bruker Tensorll. NiPc-F and NiPc-based COFs were used after they were further dried in vacuum desiccator equipped with an oil pump for 24 h under 50 °C after synthesis. The sample is positioned directly between the ATR accessory's ZnSe crystal and the ejector pin for testing. Each sample was collected 64 times from 4000 to 550 cm**^-^**^1^.

**Table S3** The assignment of peaks in ATR-IR spectra of NiPc-F ligand and NiPc-based COFs

| **Material** | **Bond and vibration type** | **Wavenumber (cm^-1^)** |
| --- | --- | --- |
| **NiPc-F** | *ν*_C-N_ | 747 |
|  | Ni-N | 883 |
|  | *ν*_C=N–C=C_ | 1093 |
|  | *ν*_C-N_ | 1346 |
|  | *ν*_C=N–C=C_ | 1410 |
|  | *ν*_C=N–C=C_ | 1613 |
| **NiPc-TAB** | *ν*_C-N_ | 747 |
|  | Ni-N | 867 |
|  | *ν*_C-N_ | 1340 |
|  | *ν*_C=N–C=C_ | 1094 |
|  | *ν*_C=N–C=C_ | 1419 |
|  | *ν*_C=N–C=C_ | 1623 |
|  | *ν*_-NH-_ | 3327 |
| **NiPc-THB** | *ν*_C-N_ | 744 |
|  | Ni-N | 863 |
|  | *ν*_C=N–C=C_ | 1094 |
|  | *ν*_C-O-C_ | 1281 |
|  | *ν*_C-N_ | 1347 |
|  | *ν*_C=N–C=C_ | 1411 |
|  | *ν*_C=N–C=C_ | 1619 |
| **NiPc-TTB** | *ν*_C-S-C_ | 704 |
|  | *ν*_C-N_ | 749 |
|  | Ni-N | 871 |
|  | *ν*_C-N_ | 1352 |
|  | *ν*_C=N–C=C_ | 1074 |
|  | *ν*_C=N–C=C_ | 1405 |
|  | *ν*_C=N–C=C_ | 1611 |

**S8 TGA curves**

Thermal gravimetric analysis (TGA) was performed using a NETZSCH TG 209F1 Libra with a 5 °C/min ramp from 30 ℃ to 800 ℃ under N_2_. Thermogravimetric analysis showed excellent thermal stability of NiPc-TTB with only a total 10% of mass loss upon heating to 400 °C. NiPc-THB and NiPc-TAB COFs show an early onset of the decomposition at around 400 °C, a total 20% around of mass loss. The higher weight loss observed in NiPc-THB and NiPc-TAB COFs could be attributed to the presence of water or guest molecules in the materials. These results indicate the thermal stability of the materials under the studied temperature range.


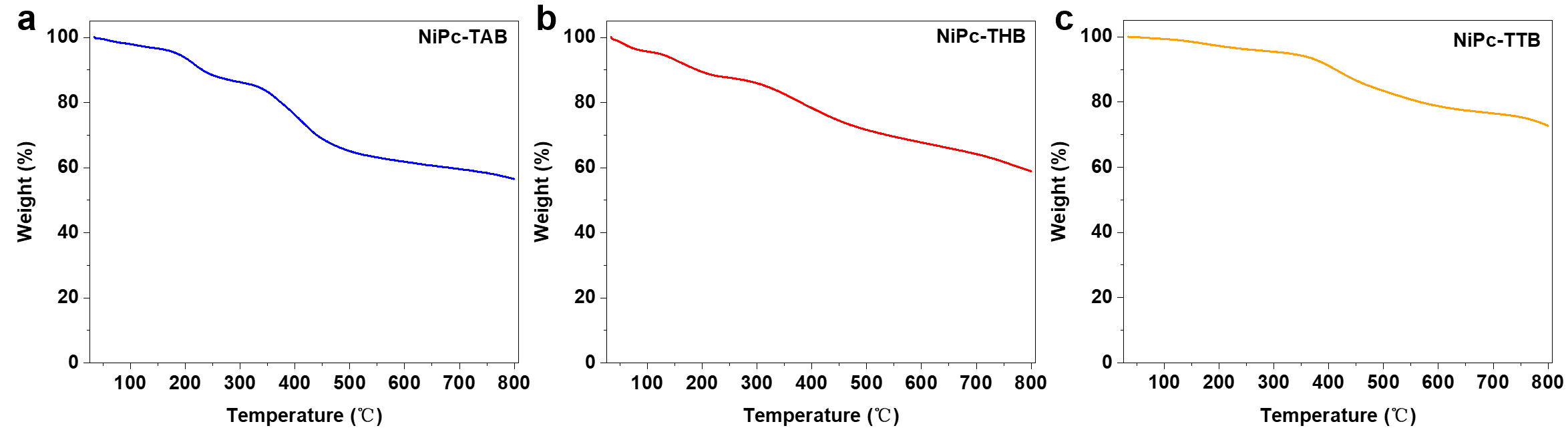


**Fig. S13** TGA curves of **a** NiPc-TAB, **b** NiPc-THB, and **c** NiPc-TTB


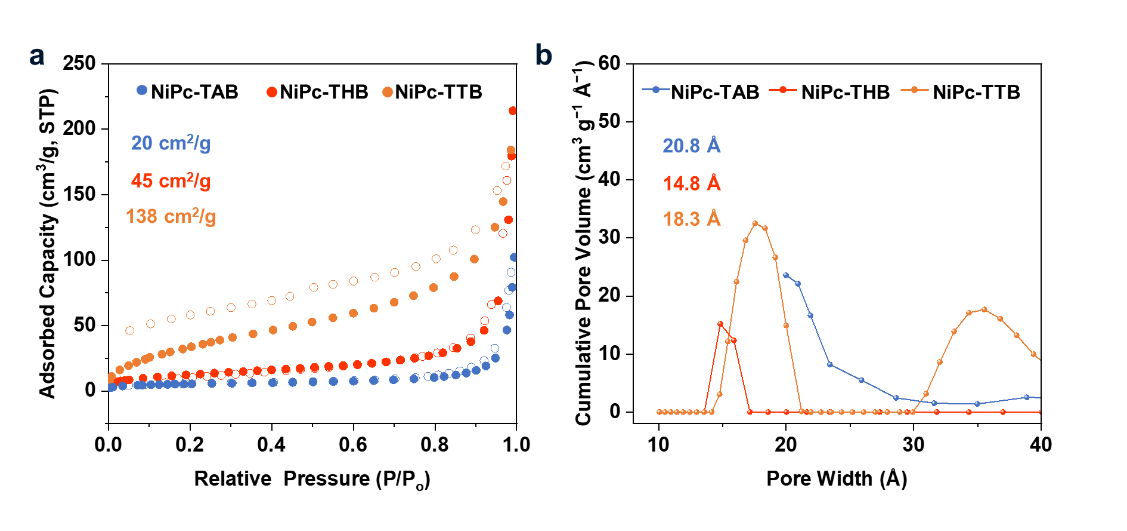


**Fig. S14** N_2_ sorption isotherms at 77 K and caculated pore size distribution for NiPc COFs

**S9 XPS spectra**

X-ray photoelectron spectroscopy (XPS) experiments of each element in this NiPc COF series before and after electrocatalysis were recorded on Thermo Scientific ESCALAB 250Xi and Thermo NEXSA G2 X-ray Photoelectron Spectrometer under ultrahigh vacuum (base pressure 10^-7^ Torr) with a pass energy of 40 eV. The measurement chamber was equipped with an excitation source of monochromatized Al (K_α_) X-ray source (*hν* = 1486.6 eV). Both survey and high-resolution spectra were obtained using a beam diameter of 400 μm. Survey spectra were obtained from 0~1100 eV to obtain elemental surface composition. Before tests, COFs samples were treated by an activation procedure in which the COFs were successively soaked into water for 3 days and ethanol for another 3 days at 50 °C, during which the solvent was changed with corresponding fresh solvent every 12 h. The COFs were further dried by an oil pump for 24 h. About 4~6 mg of each COF was mounted onto copper tape by lightly pressing. High resolution spectra were then obtained at energy regions specific to elements observed in the survey spectrum (C 1s, N 1s, O 1s, S 2p, Ni 2p).


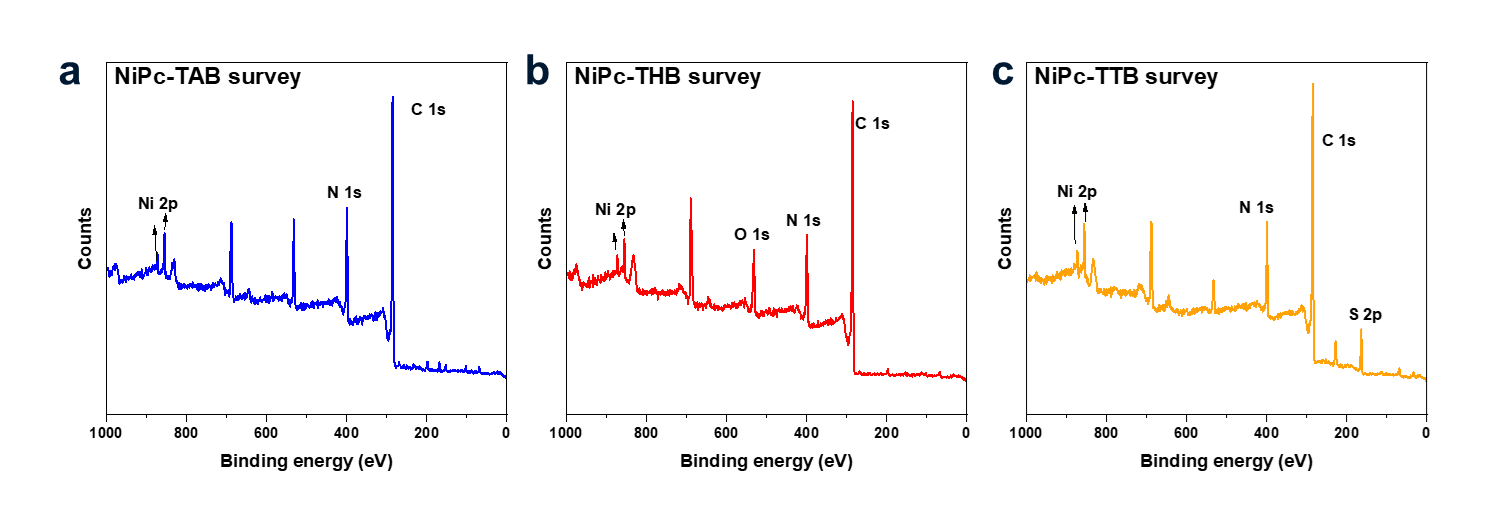


**Fig. S15** XPS survey spectra of **a** NiPc-TAB, **b** NiPc-THB, and **c** NiPc-TTB before electrocatalysis


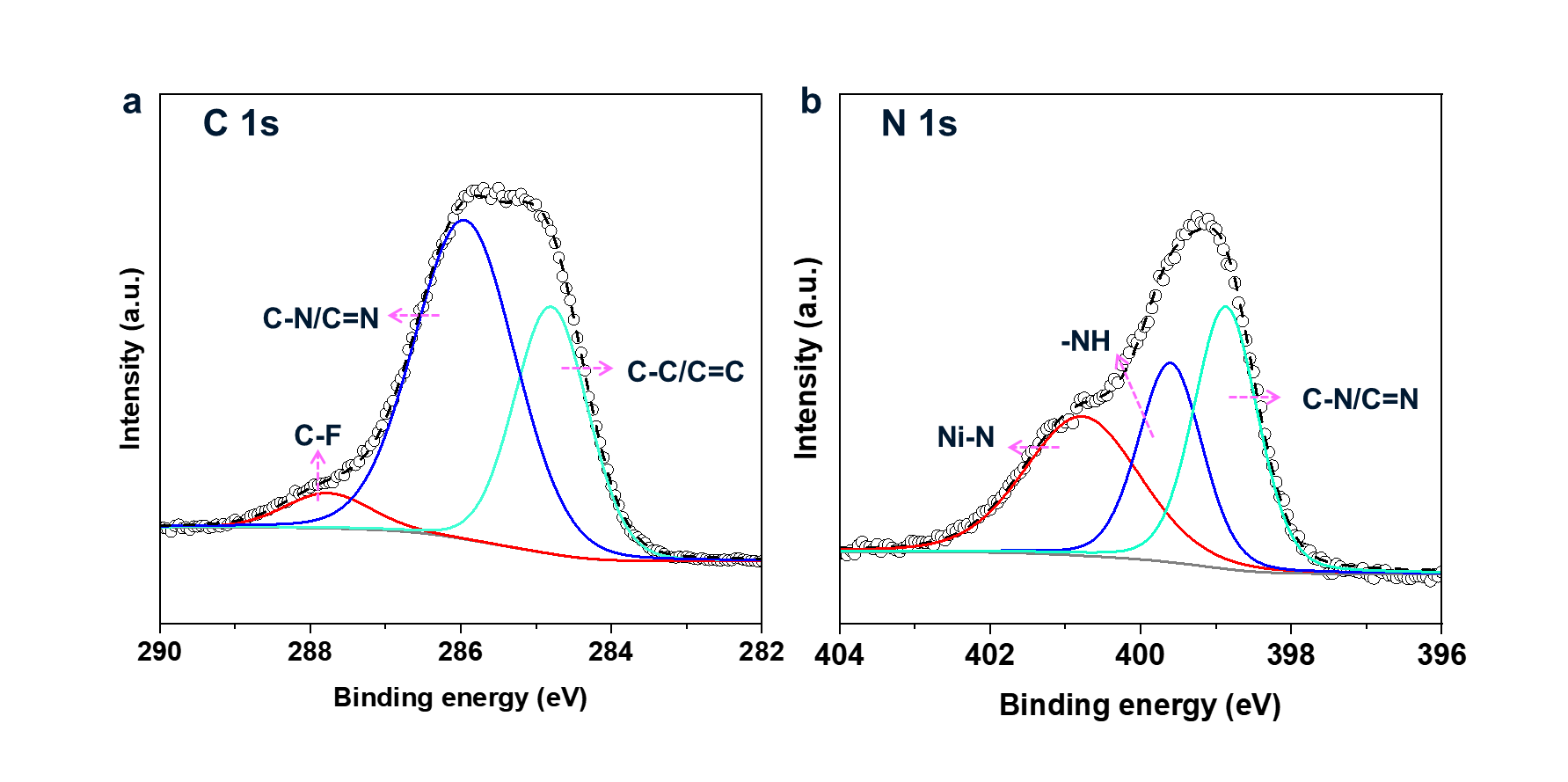


**Fig. S16** Deconvoluted XPS spectra of **a** C 1s and **b** N 1s for NiPc-TAB


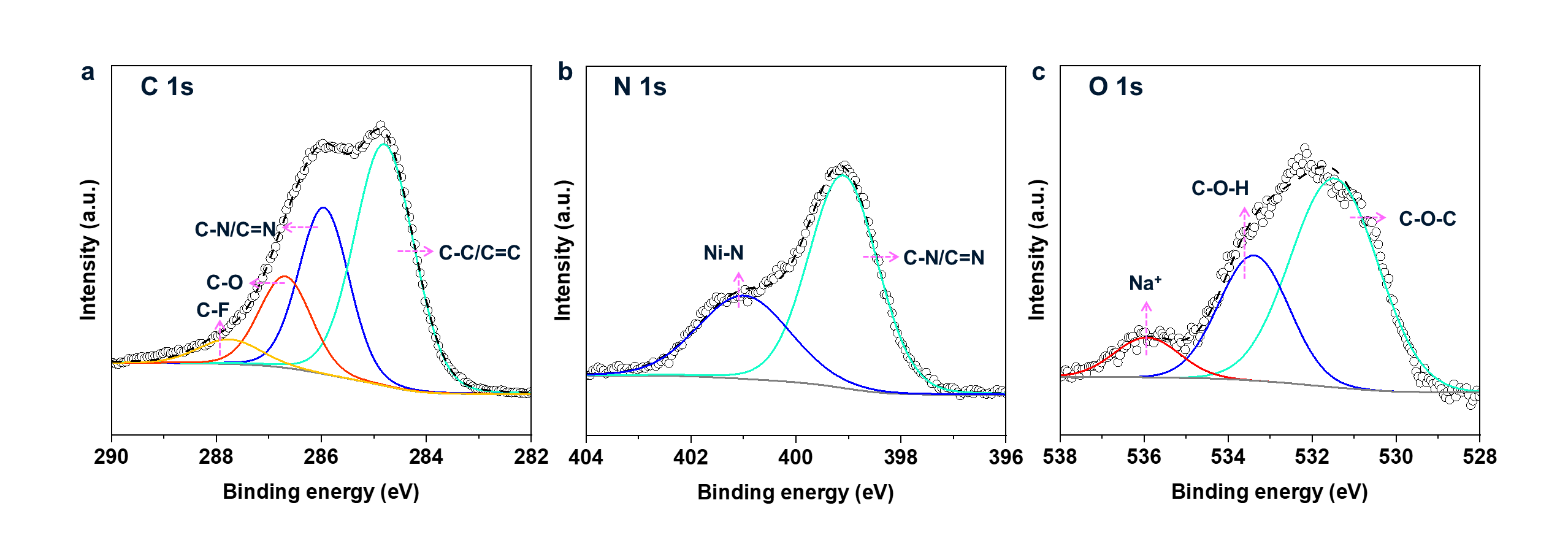


**Fig. S17** Deconvoluted XPS spectra of **a** C 1s, **b** N 1s, and **c** O 1s for NiPc-THB


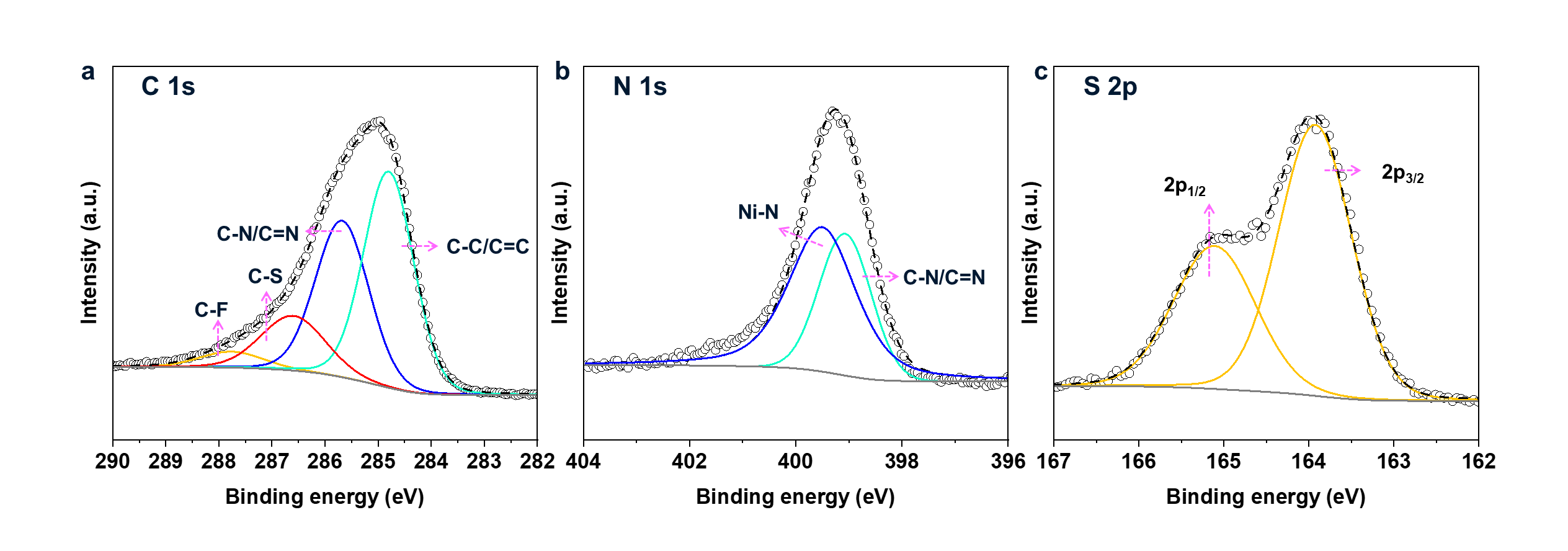


**Fig. S18** Deconvoluted XPS spectra of **a** C 1s, **b** N 1s, and **c** S 2p for NiPc-TTB

In NiPc-TAB (Fig. S16), the high-resolution spectrum of N 1s can be deconvoluted into three peaks with binding energies of 398.87, 399.60, and 400.78 eV ascribed to C=N, N-H and Ni-N [S3] in nickel phthalocyanine ligand (Fig. S16b) [S4]. In NiPc-THB (Fig. S17), the high-resolution spectrum of N 1s can be deconvoluted into two peaks with binding energies of 399.10 eV and 400.99 eV ascribed to C=N and Ni-N (Fig. S17b). High-resolution scan of O 1s region showed two peaks with binding energies at 531.47 and 533.38 eV ascribed to C-O-C and C-O-H (Fig. S17c) [S5, S6]. In NiPc-TTB (Fig. S18), the high-resolution spectrum of N 1s can be deconvoluted into two peaks with binding energies of 399.07 and 399.50 eV ascribed to C=N and Ni-N (Fig. S18b). High-resolution scan of S 2p region showed two peaks with binding energies at 163.84 eV and 165.00 eV, which can be ascribed to the formation of C-S-C (Fig. S18c).

**S10 UV−Vis−NIR spectra**

UV–Vis–NIR spectra between 200 and 2500 nm were acquired using a Shimadzu SolidSpec-3700DUV spectrophotometer at a low scan rate with a step size of 1 nm under ambient conditions. Before measuring the samples, blank baseline and zero background corrections were obtained.


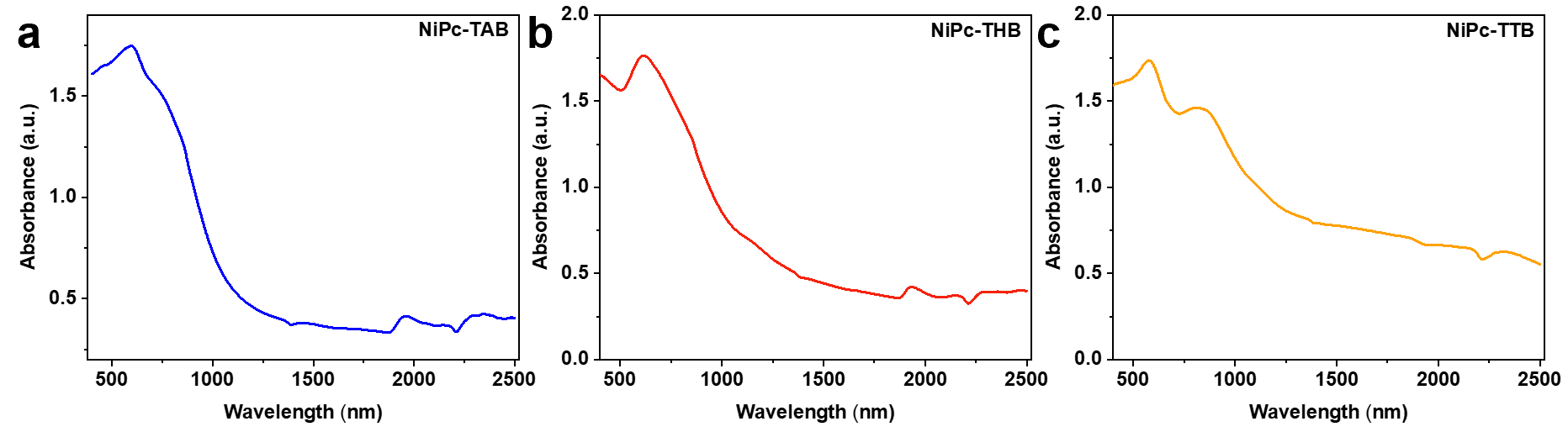


**Fig. S19** UV−Vis−NIR spectra of **a** NiPc-TAB, **b** NiPc-THB, and **c** NiPc-TTB

The Tauc method, originally developed for estimating the band gap energy of amorphous semiconductors using optical absorption spectra [S7, S8], relies on the assumption that the energy-dependent absorption coefficient *α* can be expressed by the following Eq. (S1):

$\left(\alpha\cdot h\upsilon\right)^{\frac{1}{\gamma}}=B\left(h\upsilon-E_{g}\right)$ (S1)

In the Tauc equation, which relates the absorption coefficient to the energy of incident photons, *h* represents the Planck constant, *ν* is the frequency of the photons, *E*_g_ is the optical band gap energy, and *B* is a constant. The value of the factor *γ* depends on the nature of the electron transition, with *γ* being 1/2 for direct transitions and 2 for indirect transitions in the band gap [S9-S10].


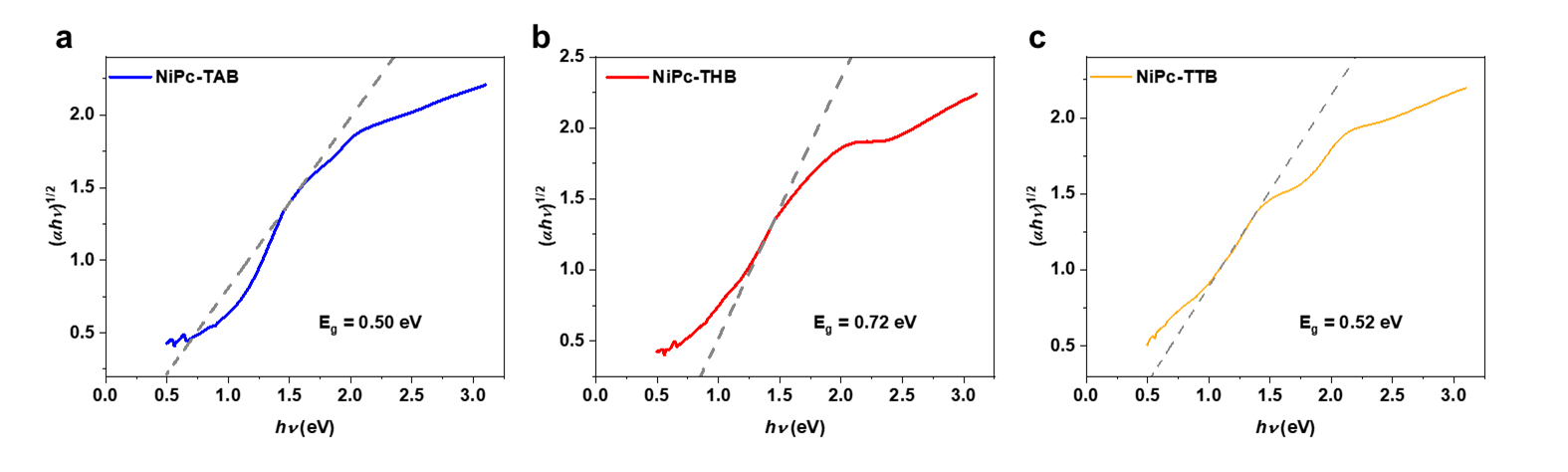


**Fig. S20** Normalized Tauc plots of **a** NiPc-TAB, **b** NiPc-THB, and **c** NiPc-TTB from the UV−Vis−NIR spectra

**S11 Conductivity test**

To make a pressed pellet, ~50 mg of the COF sample was put into a 6 mm inner-diameter split sleeve pressing die and pressed for 5 min under a pressure of approximately 1000 psi. A two-contact probe method was employed to collect bulk conductivity measurements of the COFs pellet. We calculated the bulk conductivity measurements (S/cm) using Eq. S2. Herein, L (0.15 cm) is the distance of between the probes, which equals the thickness of the pellet, A is the basal area of the pellet, V (1 volts) is the voltage of cross the probes, I (A) is current, which is measured by electrochemical analyzer.

$\sigma=\frac{I}{V}$(S2)


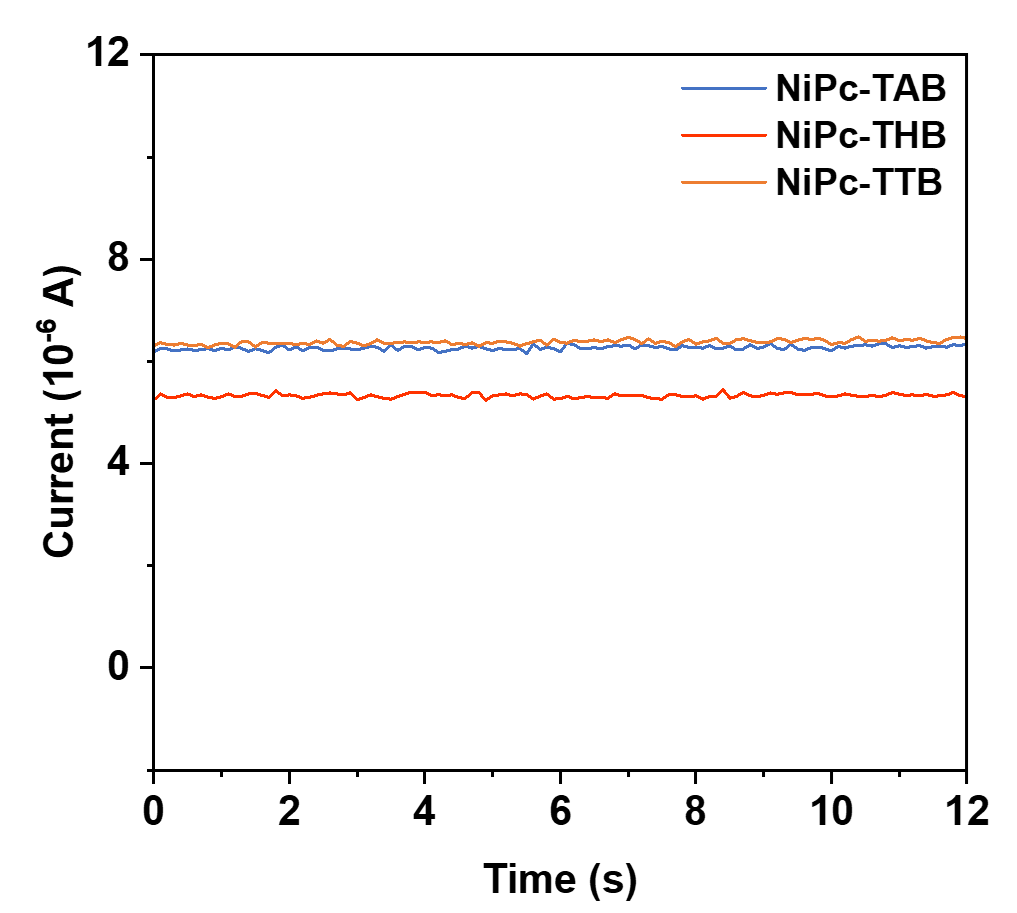


**Fig. S21** Two probe conductivity test of three NiPc COFs

**S12 Electrochemical test**

The pH of the KHCO_3_ solution (0.5 M) was measured after gently flushing the solution with N_2_ (pH = 7.2) and CO_2_ (pH = 6.8), respectively.

The overpotential *η* is the difference between the actual electrode reduction potential and the thermodynamic potential, defined by Eq. (S3):

$\eta=\left| E-E^{ϴ}\left( {{CO}_{2}}/{CO} \right) \right|$(S3)

In the equation, *E* is the applied potential (vs. RHE), and *E^ϴ^*(CO_2_/CO) is the standard electrode for CO_2_ to CO conversion which is −0.106 V (vs. RHE).

**Working electrode.** Carbon fiber paper (CFP) (HCP020P, Toray) was used as the substrate. COF inks were prepared by dispersing 4 mg of COFs and 4 mg of carbon black (Macklin, C915132, cabot vulcan xc-72R, 10-20 nm ) in a mixture of 40 μL of a Nafion 117 solution (D520 Dupont, 5 wt%) and 400 μL EtOH with the assistance of sonication for 30 min. 200 μL ink was pipetted onto CFP with a mass loading of 1.5~2 mg cm^−2^, which was further dried at room temperature for using.

**Counter electrode.** A platinum electrode was used as a counter electrode for all the electrochemical tests.

**Reference electrode.** A Ag/AgCl electrode was used as the reference electrode. All potentials were measured against Ag/AgCl electrode and converted to the potential of reversible hydrogen electrode (RHE) by using Eq. (S4):

*E*_RHE_ = 0.197V + 0.0591×pH (S4)

The reference electrode was stored under a saturated KCl solution in MiliQ water before and after using.

**Liner sweep voltammetry (LSV).** Before testing, the electrolyte solution in the working compartment was supplied with N_2_ or CO_2_ for 15 minutes. The scan rate was ranging from 50 mV s^−1^. The applied potential was ranging from −0.2 V to −1.3V (vs. RHE).

**Electrochemical impedance spectroscopy (EIS).** EIS was carried out at 100 mV alternating current voltage amplitude in a frequency range from 0.01 Hz to 100 kHz under a biased voltage of −0.8 V (vs. RHE). The setup was the same as that of the CV and chronopotentiometry.

**Cyclic voltammetry (CV).** Before testing, the electrolyte solution in the working compartment was supplied with CO_2_ for 15 minutes. The scan rate was ranging from 10 to 100 mV s^−1^.

**Electrochemical active surface area (ECSA).** ECSA was estimated by measuring the capacitive current related to double-layer capacitance from the scan-rate dependence of CVs under the potential windows of 0.2 V ~ 0.4 V (vs. RHE). The capacitance was obtained by plotting the ∆*j* = (*j*_a_ − *j*_c_) at 0.3 V (*j*_c_ and *j*_a_ are the cathodic and anodic current densities, respectively) against the scan rate. The slope (∆*j/*2*)*/(scan rate) is thus the layer capacitance.

**Chronopotentiometry.** The electrolyte solution in the working compartment was purged for 10 minutes with CO_2_ before testing. The applied potential was ranging from −0.5 V to −1.0 V (vs. RHE).

**Product quantification.** Gas products of CO_2_ electrocatalysis were detected by the gas chromatograph. Argon was used as the carrier gas. A flame ionization detector with a methanizer was used to quantify the concentration of carbon monoxide (CO). Hydrogen (H_2_) product was analyzed with a thermal conductivity detector. The peak areas were converted to gas concentration based on calibration curves.

The corresponding faradaic efficiency (FE) was calculated by Eq. (S5):

$j_{\mathrm{co}}=j_{\mathrm{product}}\times FE\times100\%$ (S5)

where the *j*_product_ and *j*_total_ are partial current density for a given gas product (CO or H_2_) and total current density, respectively.

The cathodic energy efficiency towards CO products was calculated as a descriptor of economic viability analysis by plotting each potential and current density as the horizontal coordinates, respectively, using Eq. (S6) [S11]:

${EE}_{1/2}$= $\frac{E_{CO_{2}}^{0}- E_{OER}^{0}}{E_{co_{2}R}^{applied}-E_{OER}^{0}}$ × FE_CO_ × 100% (S6)


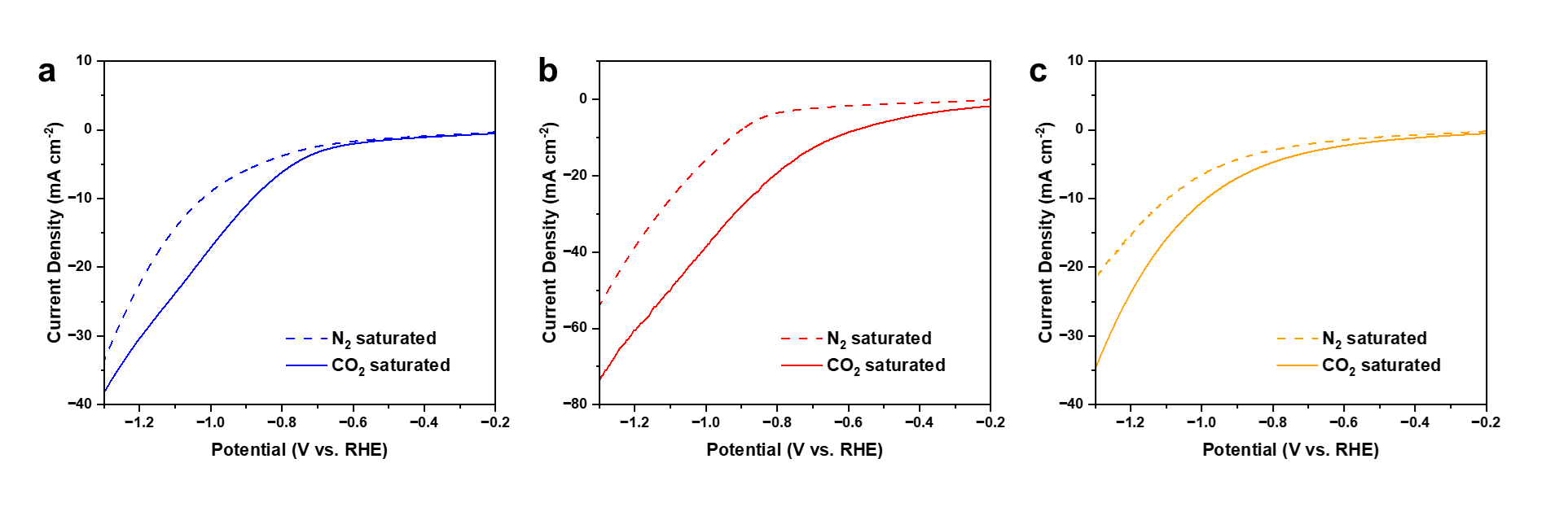


**Fig.** **S22** LSV curves for **a** NiPc-TAB, **b** NiPc-THB, and **c** NiPc-TTB in a CO_2_-saturated (solid line, pH = 6.8) and N_2_-saturated (dashed line, pH = 7.2) KHCO_3_ solution

NiPc-TAB

NiPc-THB

NiPc-TTB

**Fig. S23** ^1^H NMR spectra for electrolytes after electrolysis at all potentials. From top to bottom: NiPc-TAB, NiPc-THB, and NiPc-TTB

**
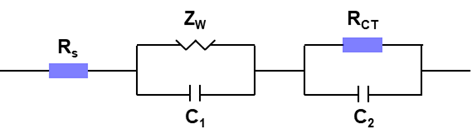
**

**Fig. S24** Equivalent circuit used for fitting the data. In the circuit, R_S_ shows the combination of the resistance of electrodes and electrolyte. A diffusion element Z_W_ represents the diffusion of CO_2_ in the electrolyte. C_1_ is the capacitance value of working electrode-electrolyte interface. C_2_ and R_CT_ are the capacitance and charge transfer resistance values of the counter electrode-electrolyte interface, respectively. Z_W_ and R_CT_ are in parallel with the capacitive elements C_1_ and C_2_

**Table S4** The resistance and capacitance values for the fitting equivalent circuit for three NiPc COFs

| **Values**  **COFs** | **R_S_ (Ω)** | **R_CT1_ (Ω)** | **Z_WR_**  **(Ω)** | **Z_WT_**  **(Ω)** | **Z_WP_**  **(Ω)** | **C_1_ (F)** | **C_2_ (F)** |
| --- | --- | --- | --- | --- | --- | --- | --- |
| **NiPc-TAB** | 3.479 | 12.64 | 8.79 | 1.498 | 0.3596 | 1.8683 × 10^-4^ | 8.4099 × 10^-3^ |
| **NiPc-THB** | 2.777 | 2.32 | 5.379 | 0.90725 | 0.30256 | 1.3223 × 10^-3^ | 7.9944 × 10^-3^ |
| **NiPc-TTB** | 2.9891 | 32.57 | 12.83 | 0.21591 | 0.54327 | 2.1442 × 10^-3^ | 1.5267 × 10^-3^ |


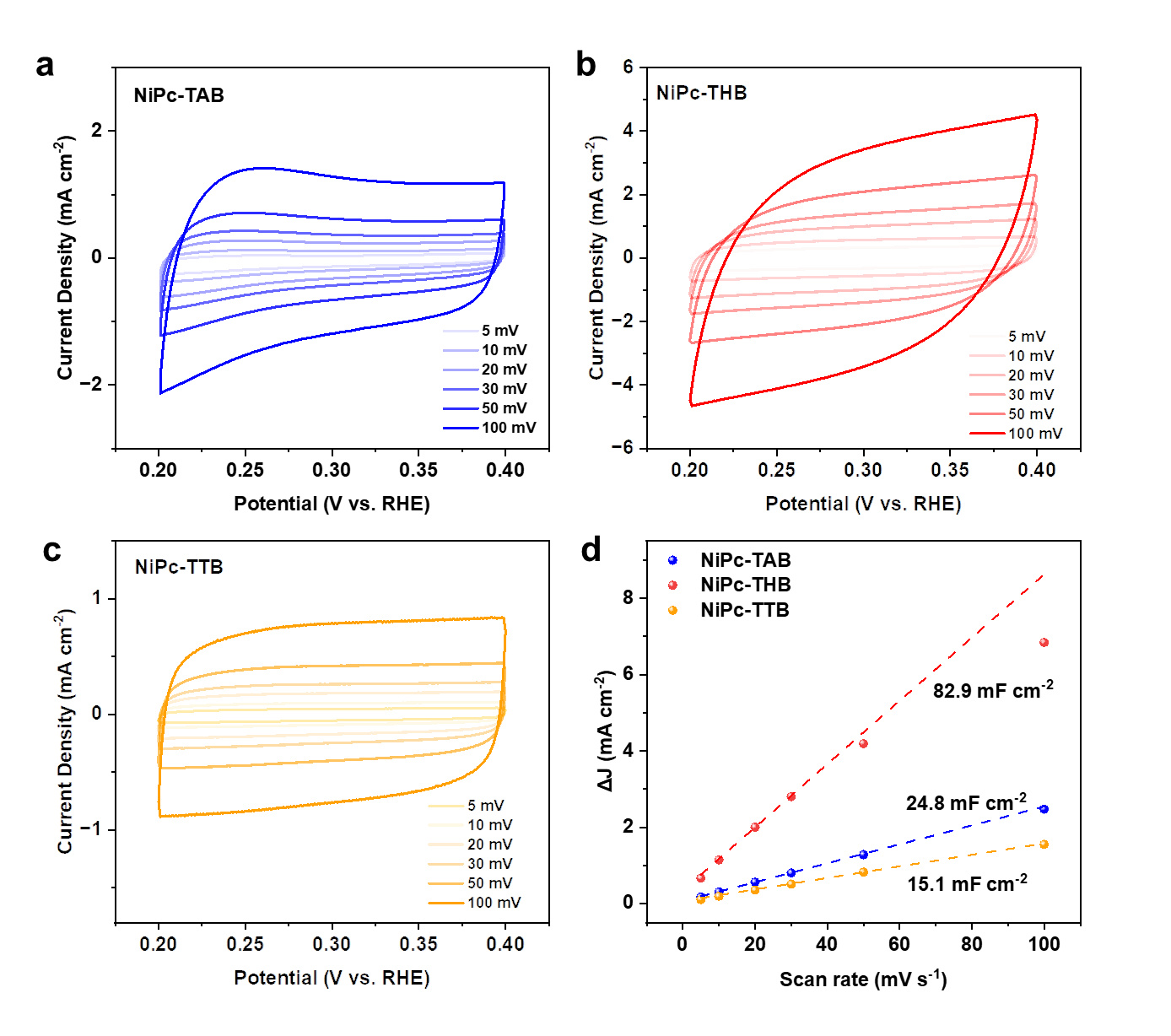


**Fig. S25** CV curves of **a** NiPc-TAB, **b** NiPc-THB, and **c** NiPc-TTB at different scan rates. **d** Capacitive current density against the scan rate of NiPc-based COFs


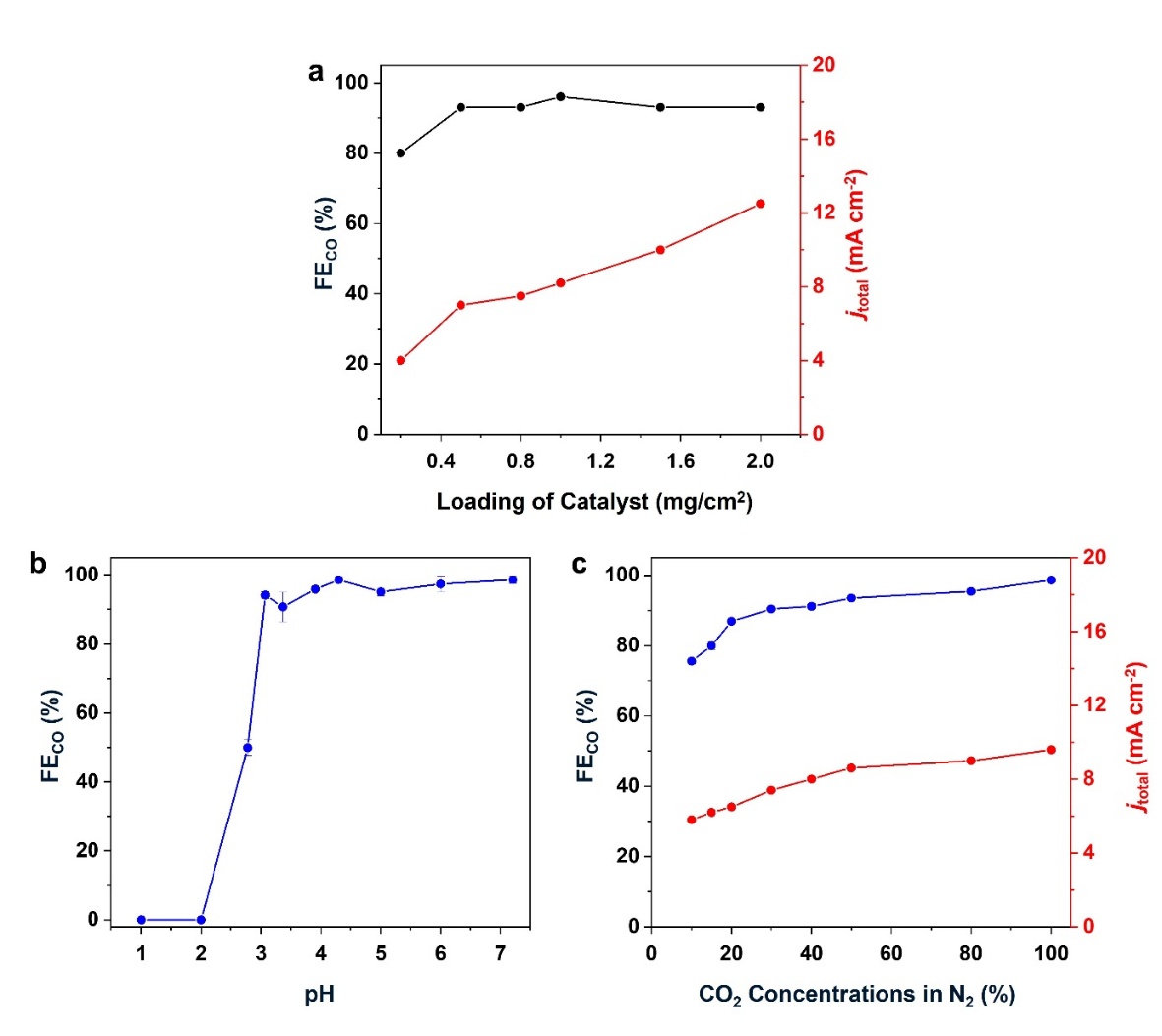


**Fig. S26** The Faradaic efficiency for the electrocatalytic CO_2_RR of NiPc-TAB under different loadings in an H-cell at −0.8 V (vs. RHE). Increasing the loadings of catalyst over 0.5 mg cm^−2^ resulted in no obvious change of CO selectivity while giving an increased current density which might be due to the increased available catalytic sites with more catalyst


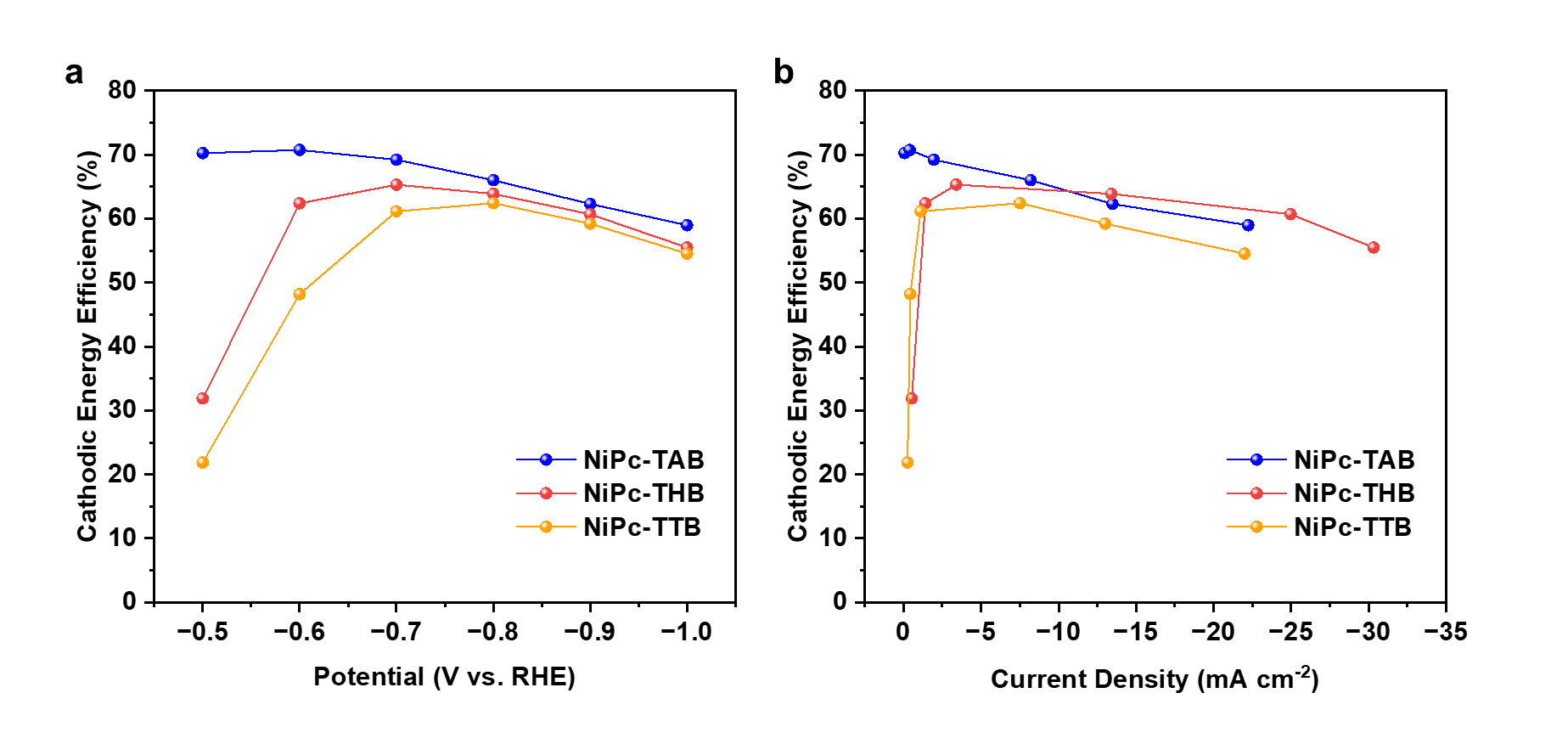


**Fig. S27** Comparison of cathodic energy efficiency over NiPc-TAB, NiPc-THB, and NiPc-TTB at **a** each potential and **b** current density, respectively


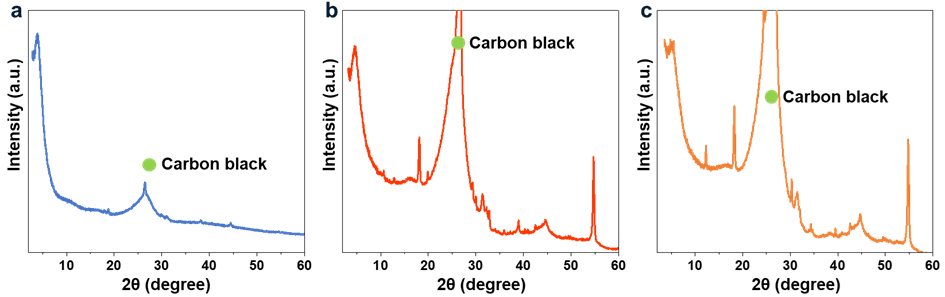


**Fig. S28** PXRD patterns of **a** NiPc-TAB, **b** NiPc-THB, and **c** NiPc-TTB sample after electrocatalytic CO_2_RR. The presence of additives in COF catalysts and the irregular residual guests, such as KHCO_3_, Nafion in the pores, may lead to weakened peak intensities in PXRD compared with pristine COFs


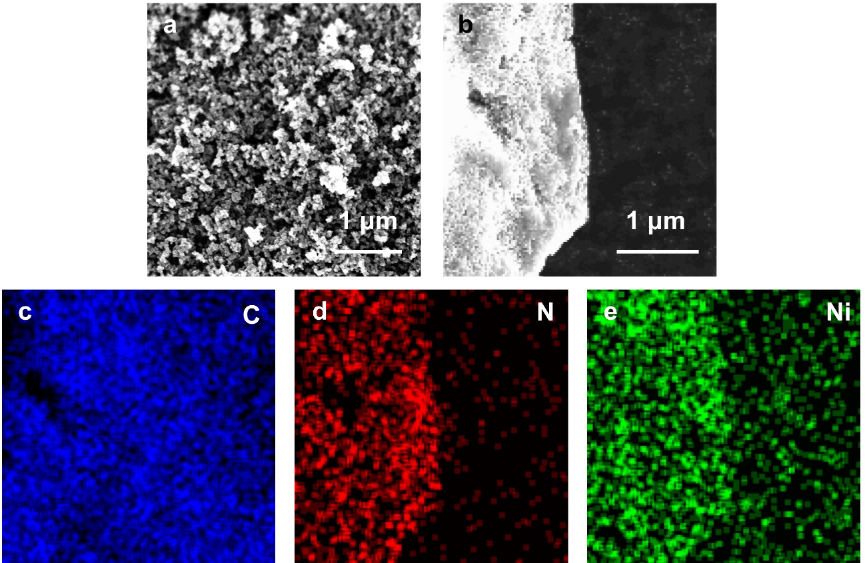


**Fig. S29** **a** SEM images of NiPc-TAB. **b** SEM images and **c-f** corresponding elemental mappings of the C, N, and Ni elements, respectively, for NiPc-TAB after electrocatalytic CO_2_RR

**
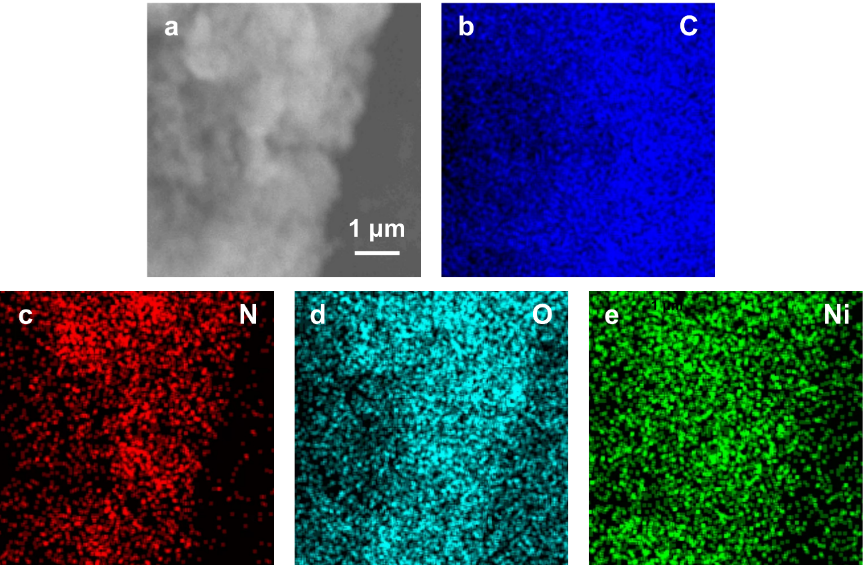
**

**Fig. S30** **a** SEM images and **b-d** corresponding elemental mapping of the C, N, O, and Ni elements, respectively, for NiPc-THB after electrocatalytic CO_2_RR

**
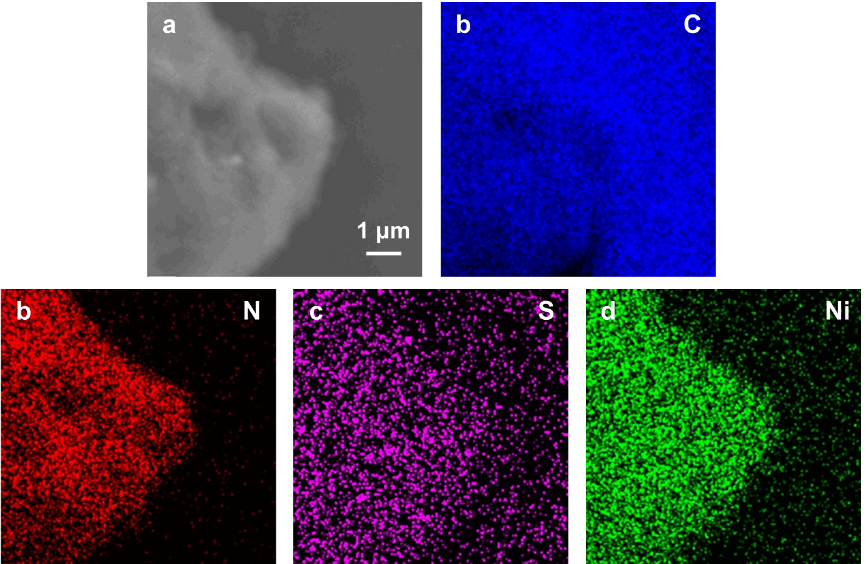
**

**Fig. S31** **a** SEM images and **b-d** corresponding elemental mapping of the C, N, S, and Ni, respectively, for NiPc-TTB after electrocatalytic CO_2_RR


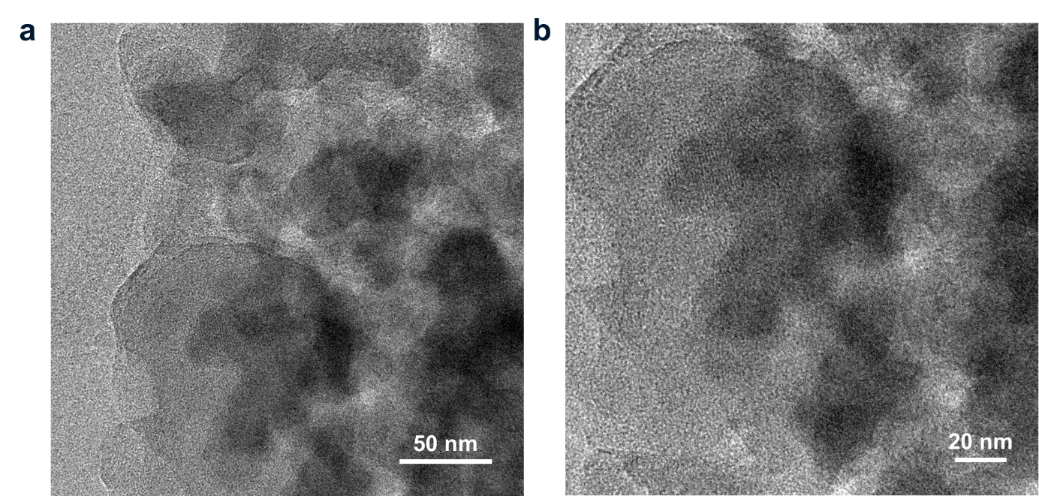


**Fig. S32** TEM images of NiPc-TAB at different magnifications after electrocatalytic CO_2_RR


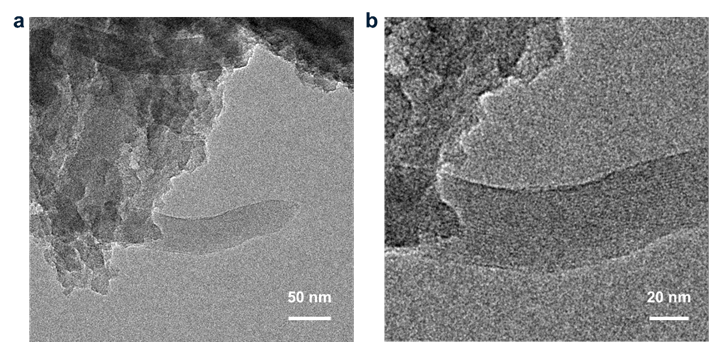


**Fig. S33** TEM images of NiPc-THB at different magnifications after electrocatalytic CO_2_RR

**
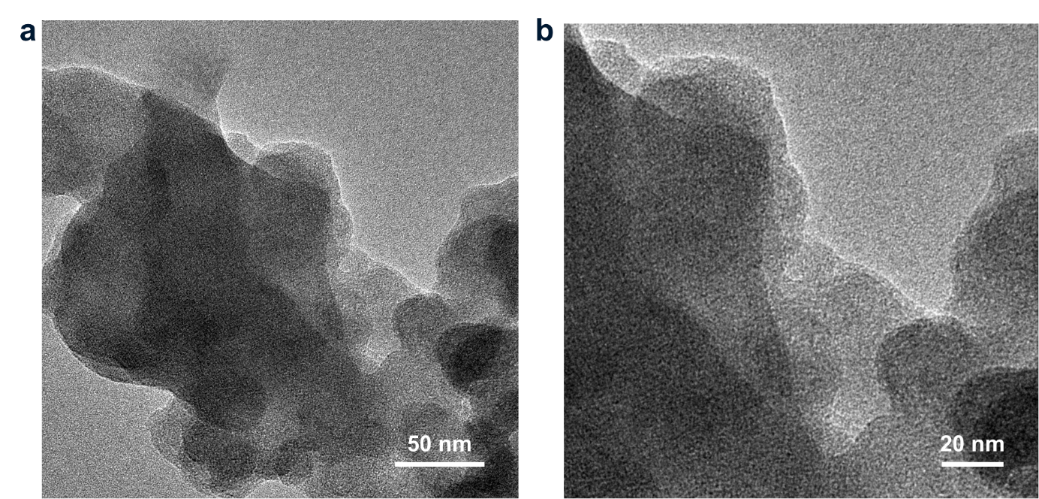
**

**Fig. S34** TEM images of NiPc-TTB at different magnifications after electrocatalytic CO_2_RR


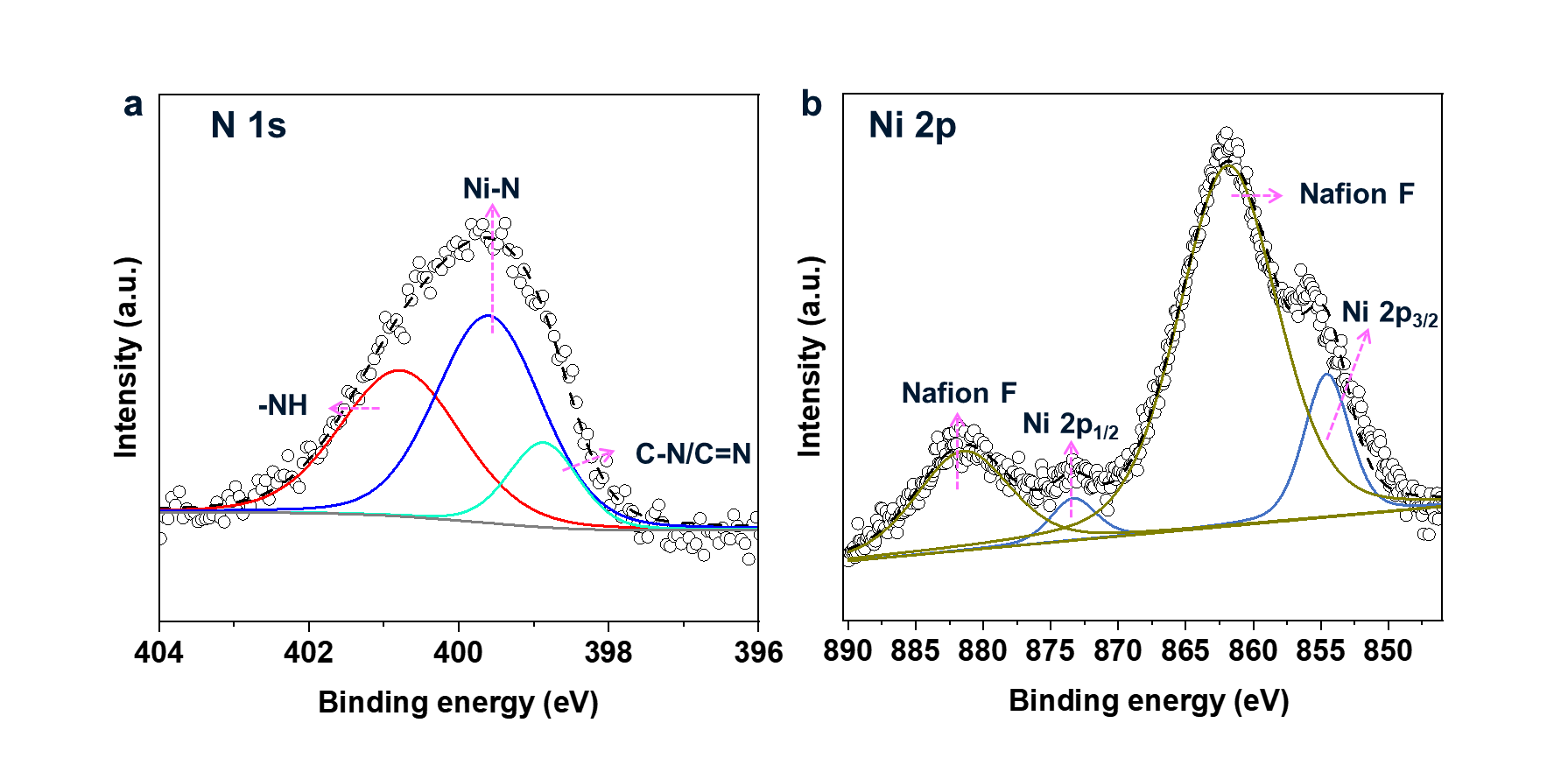


**Fig. S35** Deconvoluted XPS spectra of **a** N 1s and **b** Ni 2p for NiPc-TAB after electrocatalytic CO_2_RR


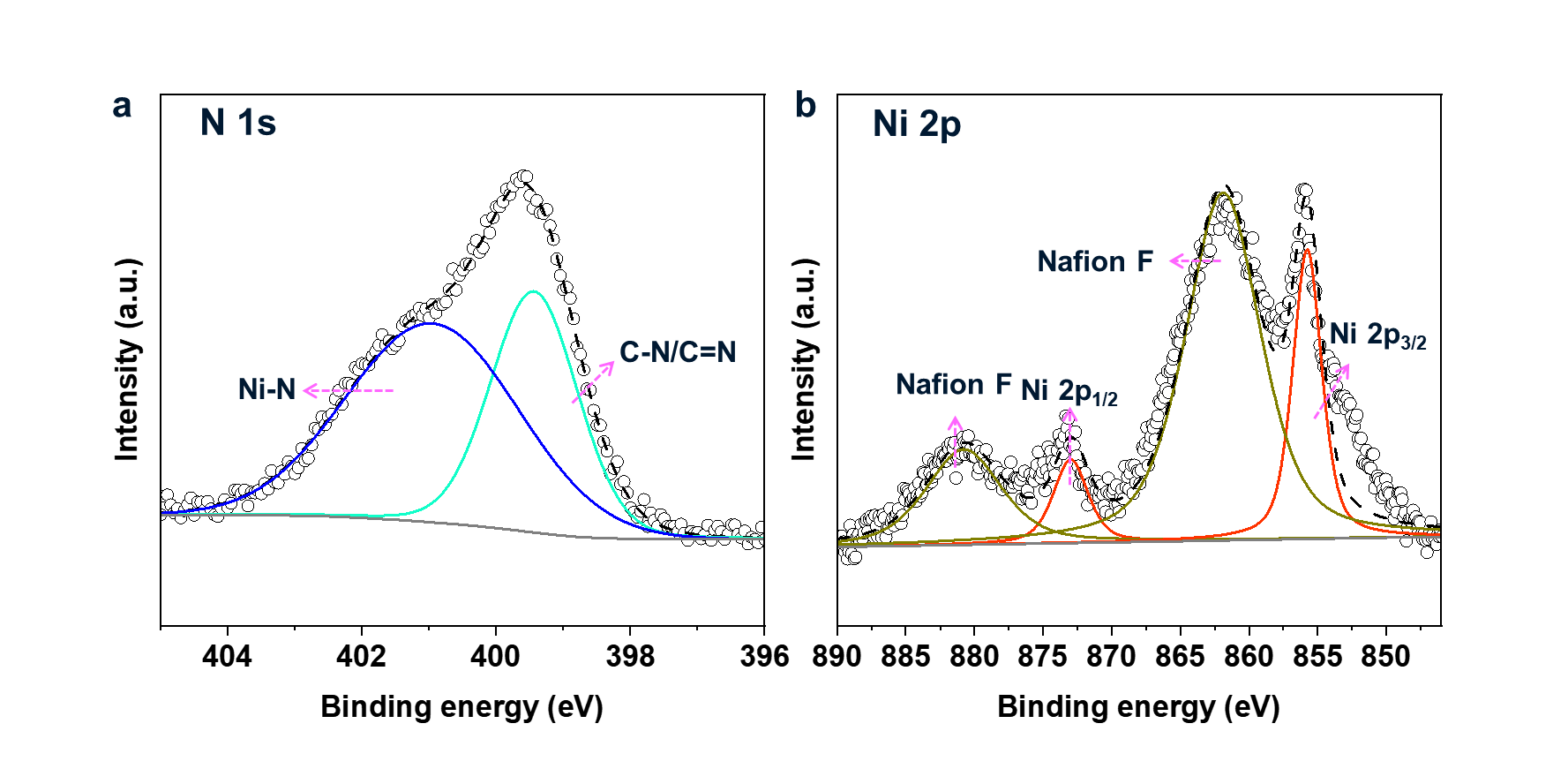


**Fig. S36** Deconvoluted XPS spectra of **a** N 1s and **b** Ni 2p for NiPc-THB after electrocatalytic CO_2_RR


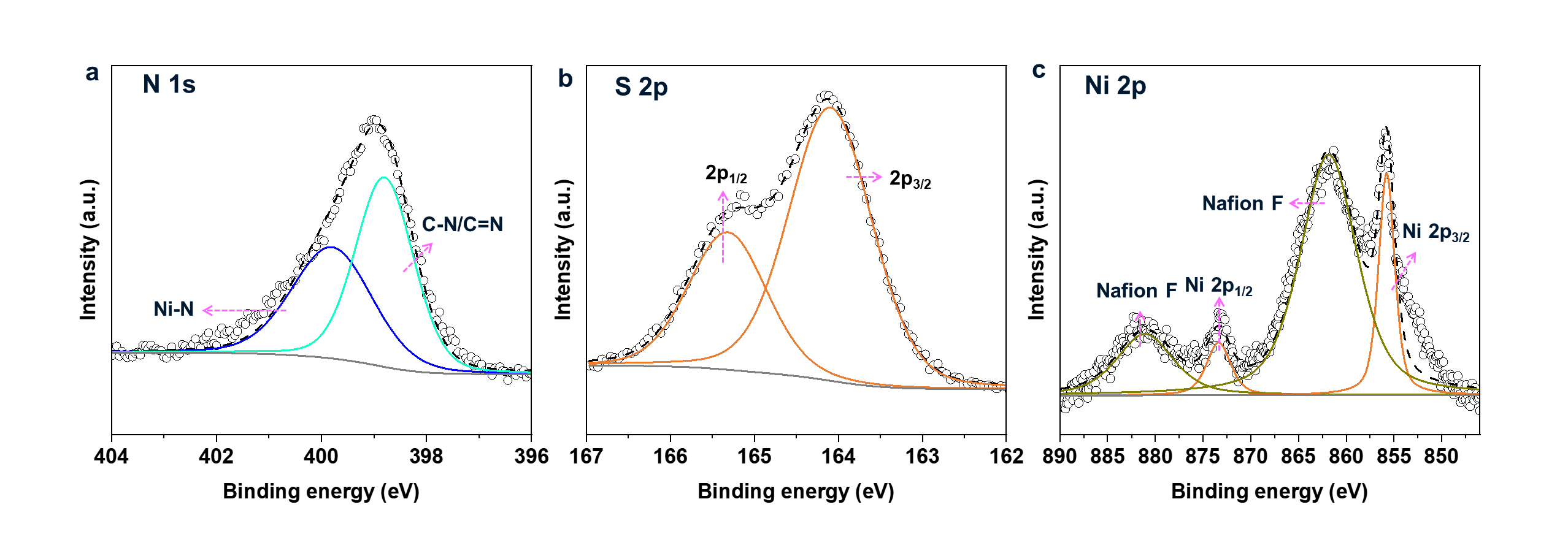


**Fig. S37** Deconvoluted XPS spectra of **a** N 1s, **b** S 2p, and **c** Ni 2p for NiPc-TTB after electrocatalytic CO_2_RR

**S13 Performance comparison**

We have summarized the performance matrix of the catalytic electrochemical conversion of CO_2_ to CO by the utilization of several important classes of the materials, including reticular materials, molecular metallophthalocyanine (MPc), metalloporphyrin (MPy), and the composites bases on molecular MPc and MPy. The corresponding data are listed in Table S5.

**Table S5** Comparison of the electrochemical reduction of CO_2_ to CO catalyzed by MPc based COFs used in this work with reticular materials and other related materials

| **Type of catalysts** | Electrocatalyst | E  (V vs. RHE) | FE_CO_  (%) | *j*  (mA cm^−2^) | *Cathodic EE (%)* | Tafel slopes (mV dec^−1^ ) | Ref. |
| --- | --- | --- | --- | --- | --- | --- | --- |
| **MPc and MPy based reticular materials** | NiPc-TAB | −0.80 | 99.90 | −8.2 | 65.94 | 134.8 | this work |
|  | NiPc-THB | −0.80 | 93.00 | −13.4 | 61.39 | 190.7 | this work |
|  | NiPc-TTB | −0.8 | 90 | −7.5 | 59.41 | 176.3 | this work |
|  | NiPc-TFPN COF | −0.9 | 99.80 | −14.1 | 62.78 | 209.9 | [S12] |
|  | NiPc-NH-TFPN-NH_2_ | −0.8 | 99.6 | −24.33 | 65.75 | 103 | [S13] |
|  | CoPc-DSDSCOF | −0.9 | 96.5 | −26 | 60.71 | 242 | [S14] |
|  | TFPc-PBBA-COF | −0.9 | 97 | −25 | 61.02 | 226.2 | [S15] |
|  | CoPc-PI-COF-1 | −0.7 | 93 | −9.4 | 64.57 | 95 | [S16] |
|  | CoPc-PDQ-COF/CB | −0.66 | 96 | −22.2 | 68.06 | 112 | [S17] |
|  | CoPc-2H_2_Por COF | −0.55 | 95 | <−6 | 71.52 | 123 | [S18] |
|  | COF-367-Co | −0.67 | 90 | −3.3 | 63.47 | over 470 | [S19] |
|  | MOF-1992 | −0.63 | 80 | −16.5 | 57.63 | N/A | [S20] |
|  | CuPcF_8_-CoNPc-COF | −0.62 | 97 | −16.5 | 70.26 | 104 | [S21] |
|  | NiPcF_8_-NiPc-COF | −0.87 | 93 | N/A | 59.34 | N/A | [S21] |
|  | TTF-Por(Co)-COF | −0.7 | 95 | −6.88 | 65.96 | N/A | [S22] |
|  | pCoNiPc | −0.8 | 94 | −16 | 62.05 | ~118 | [S23] |
|  | Al_2_(OH)_2_TCPP-Co | −0.70 | 90 | −1.0 | 62.49 | 165 | [S24] |
|  | NiPc-NiO_4_ | −0.84 | 98.4 | −34.5 | 63.70 | N/A | [S25] |
|  | Re-SURMOF | −1.60 | 93 | −2.5 | 44.04 | N/A | [S26] |
|  | MOF-545(Fe)/CB | −0.60 | 91 | −1.2 | 66.63 | 188 | [S27] |
|  | Fe-PB | −0.63 | 85.00 | 0.20 | 61.24 | 165 | [S28] |
|  | PcCu-O_8_-Zn/CNT (1:0.5) | −0.70 | 88.00 | −4.0 | 61.10 | 125 | [S29] |
|  | Co-TTCOF | −0.70 | 91.30 | −1.84 | 63.39 | 237 | [S30] |
| **Molecular MPc and MPy** | FTDHPP | −0.58 | 94.00 | −0.31 | 69.59 | N/A | [S31] |
|  | Cobalt protoporphyrin | −0.80 | 37.40 | −0.33 | 24.69 | N/A | [S32] |
|  | CoFPc | −0.80 | 93.00 | −4.2 | 61.39 | 270 | [S33] |
|  | Cu(II)-5,10,15,20-tetrakis-(2,6-dihydroxyphenyl)porphyrin | −0.98 | 10.00 | <−2 | 6.06 | N/A | [S34] |
|  | Zinc(II) 5,10,15,20-tetramesitylporphyrin | −1.94 | 95.00 | −2.1 | 40.16 | N/A | [S35] |
|  | CoPc | −0.80 | 99.00 | −8 | 65.35 | ~121 | [S36] |
| **MPc and MPy based composite** | Co chlorin/CNTs | −1.10 | 64.00 | N/A | 36.81 | N/A | [S37] |
|  | CoPc/poly-4-vinylpyridine | −0.73 | 89.00 | −2 | 60.85 | N/A | [S38] |
|  | CAT_Pyr_/CNT | −0.59 | 93.00 | −0.24 | 68.47 | N/A | [S39] |
|  | CoPc/CNT | −0.63 | 92.00 | −10 | 66.28 | N/A | [S40] |
|  | CoPPc/CNT | −0.61 | 80.00 | −20 | 58.26 | 121 | [S36] |
|  | Co-TPP/CNT | −0.50 | 83.00 | −0.59 | 64.29 | 255 | [S41] |
|  | Protoporphyrin IX cobalt chloride CoPP@CNT | −0.65 | 98.30 | −21 | 70.06 | 118 | [S42] |
|  | CoPc/CNT | −0.78 | 95.00 | −11 | 63.33 | N/A | [S43] |
|  | CoPc-NH_2_/CNT | −0.78 | 95.00 | −12.5 | 63.33 | N/A | [S43] |
|  | CoPc@carbon powder | −0.68 | 93.00 | −18.1 | 65.25 | N/A | [S44] |
|  | CoCoPcP/CNTs | −0.44 | 94.00 | −8.1 | 75.43 | 123 | [S45] |


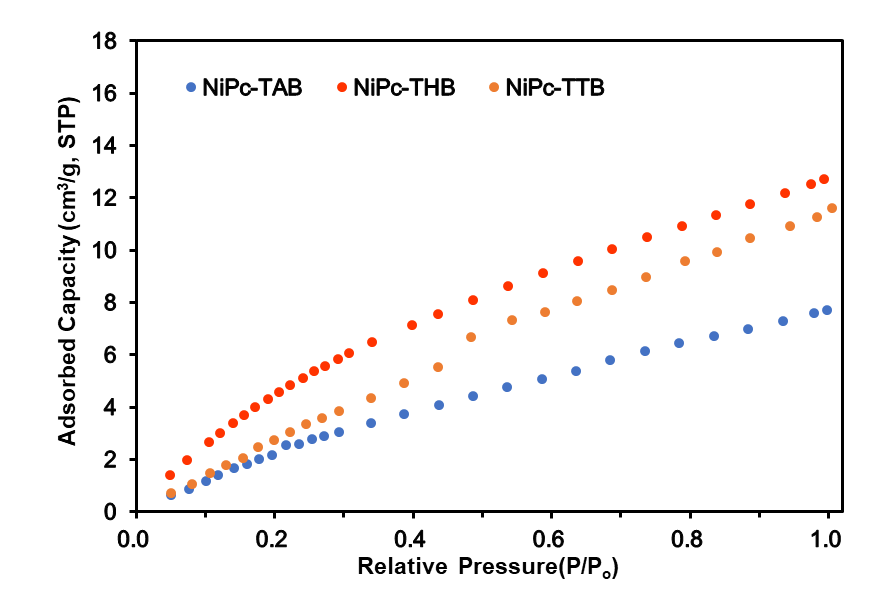


**Fig. S38** CO_2_ sorption isotherms of NiPc-based COFs

**S14 Computational study of electronic properties**

For the computation of electronic properties including band structures and density of states (DOS), the functional GGA with PBE was employed with energy cutoffs set at 435.4 eV, 571.4 eV, and 435.4 eV for NiPc-TAB, NiPc-THB, and NiPc-TTB, respectively. The Brillouin zones are sampled using a 2 × 2 × 4 k-point mesh in the Monkhorst-Pack scheme.


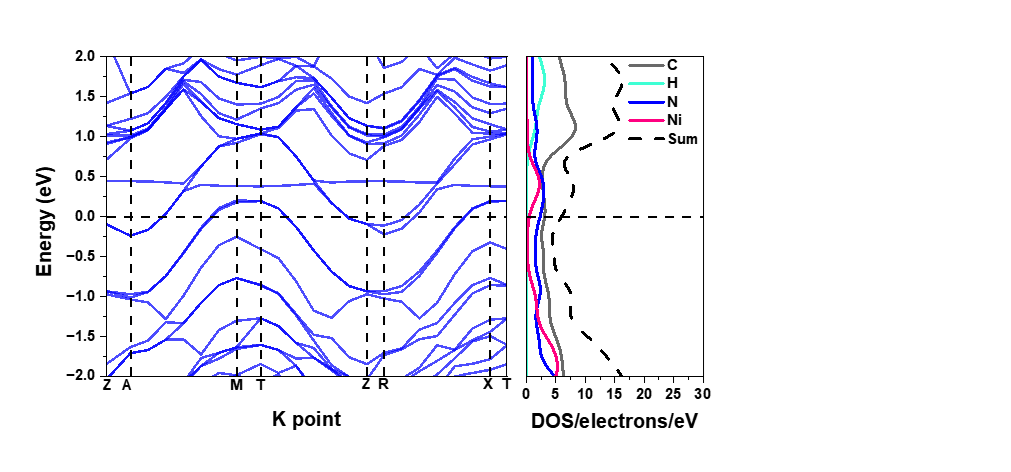


**Fig. S39** Calculated electronic band structure (left) and DOS (right) for NiPc-TAB


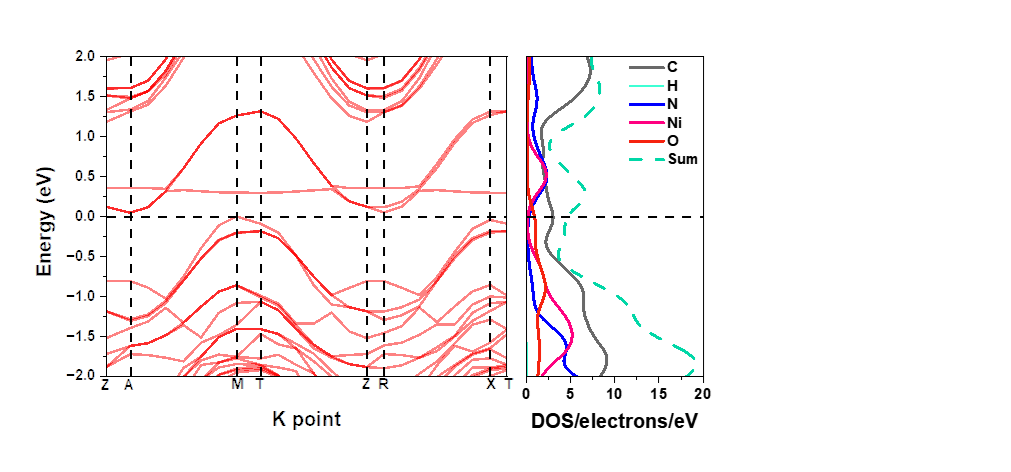


**Fig. S40** Calculated electronic band structure (left) and DOS (right) for NiPc-THB


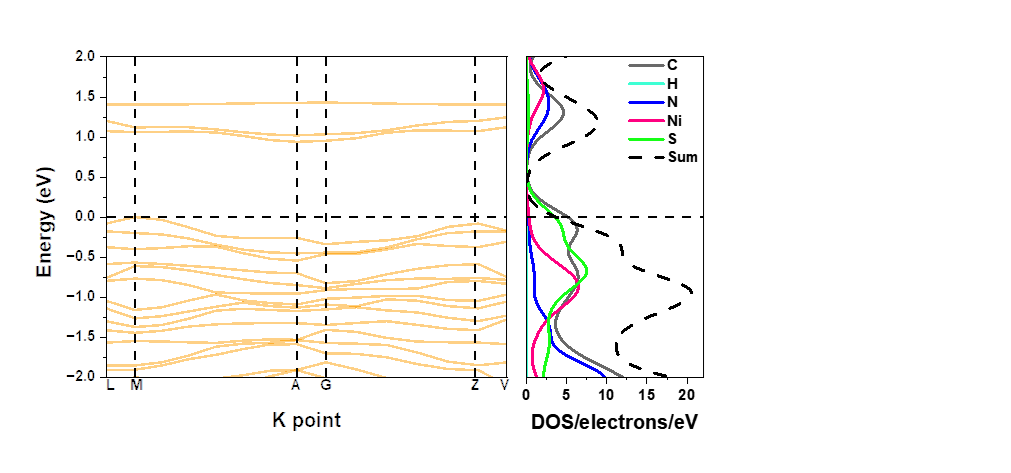


**Fig. S41** Calculated electronic band structure (left) and DOS (right) for NiPc-TTB

**
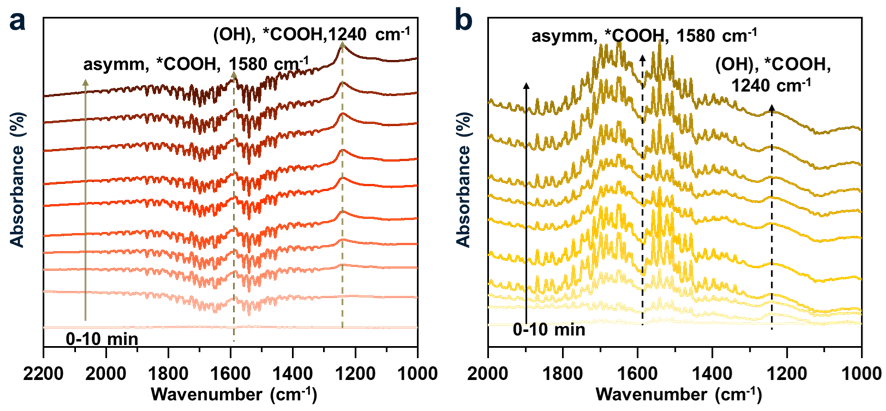
**

**Fig. S42** *In-situ* FTIR spectra of **a** NiPc-THB and **b** NiPc-TTB collected at −0.80 V (vs*.* RHE) under CO_2_-saturated 0.5 M KHCO_3_ electrolyte

**S15 Computational investigation of catalytic mechanism**

**Computational details**. Electrocatalytic CO_2_RR reduction is a proton-coupled electron transfer process.

CO_2_(g) + 2H^+^ (aq) + 2e^-^(aq) → CO(g) + H_2_O (S6)

In the calculation, following four steps are considered in electrocatalytic carbon dioxide

reduction:

M + CO_2_ + 2H^+^ + 2e^-^ → [M-CO_2_] + 2H^+^+ 2e^-^ (S7)

[M-CO_2_] + 2H^+^ + 2e^-^→ [M-COOH] + 2H^+^+ 2e^-^ (S8)

[M-COOH] + H^+^ + e^-^ → [M-CO] + H_2_O (S9)

[M-CO] + H_2_O → M + CO + H_2_O (S10)

Electronic calculations were performed to optimize the geometry and calculate the Gibbs free energy of all intermediates involved in the reactions using density functional theory (DFT) in the Dmol3 module of Materials Studio.

$G=H-TS=E_{\mathrm{DFT}}+E_{\mathrm{ZPE}}+\int_{0}^{298.15} C_{p}dT-TS$ (S11)

$\mu\left( H^{+} \right) + \mu\left( e^{-} \right)=\frac{1}{2}\mu\left[ H_{2}\left( g \right) \right]$(S12)

In eq S10, T is the system temperature (298.15 K), E_DFT_ is the electron energy calculated by DFT above, E_ZPE_ is the zero-point energy, which is a combination of vibrational, translational, and rotational energy at absolute zero, C_p_ is the heat capacity, and S is entropy. E_ZPE_, C_p_, and S are calculated using the harmonic approximation of the frequency analysis in the Dmol3 module.

|  | [Ni-CO_2_] | | [Ni-COOH] | | [Ni-CO] | |
| --- | --- | --- | --- | --- | --- | --- |
| NiPc-TAB | 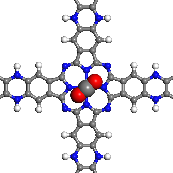 | 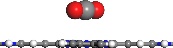 | 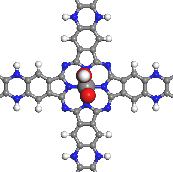 | 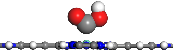 | 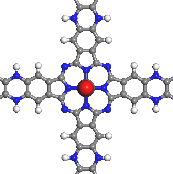 | 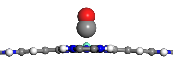 |
| NiPc-THB | 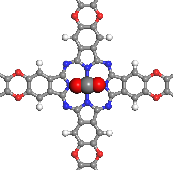 | 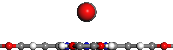 | 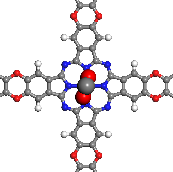 | 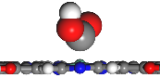 | 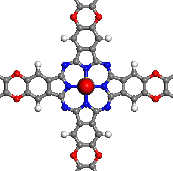 | 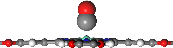 |
| NiPc-TTB | 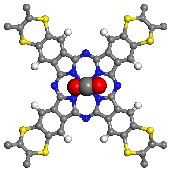 | 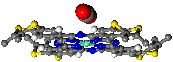 | 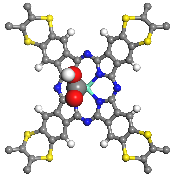 | 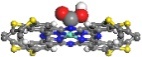 | 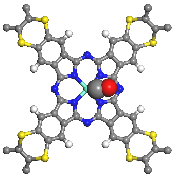 | 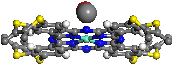 |

**Fig. S43** Optimization of the ground state structure of NiPc-TAB, NiPc-THB, and NiPc-TTB monolayer by CO_2_ adsorption, COOH, and CO adsorbed on the NiPc-TAB, NiPc-THB, and NiPc-TTB monolayer. Using the Dmol3 module in Materials Studio, the GGA-PBE exchange correlation function and DND base set were used to optimize the structure


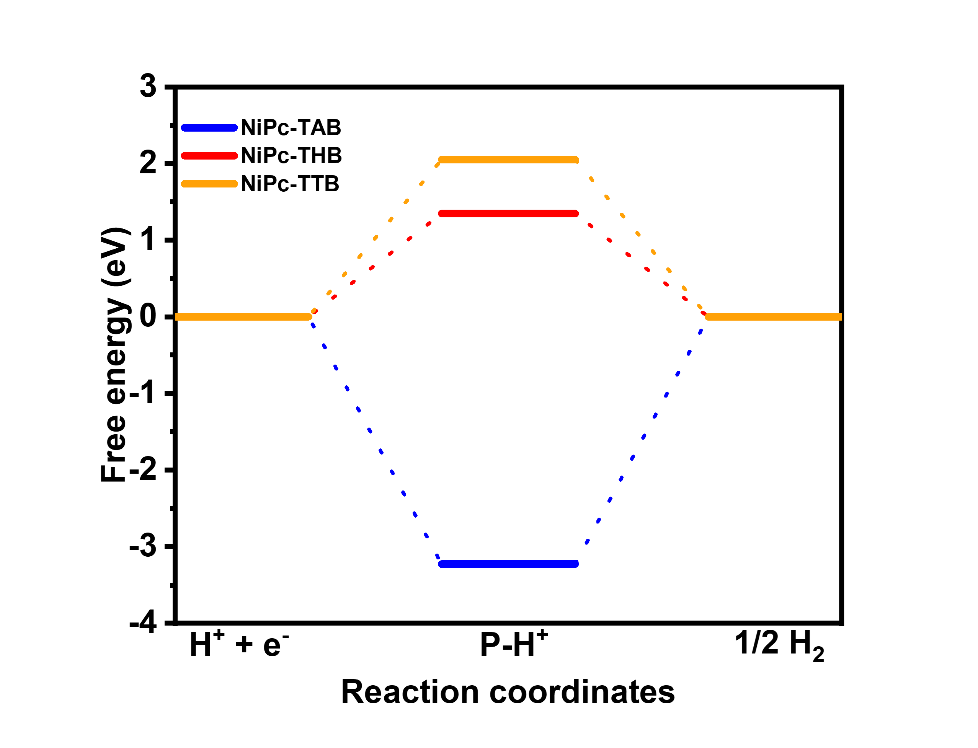


**Fig. S44** Free energy diagrams of HER for NiPc-based COFs

**Table S6** Calculated electronic energies (E_DFT_), zero-point vibrational energies (E_ZPE_), entropies (TS), thermal corrections ($\int C_{p}\mathrm{dT}$), and free energies (G) associated with NiPc-TAB catalyzed process

|  | **E_DFT_**  **(Ha)** | **E_ZPE_**  **(kcal mol^-1^)** | **-TS**  **(cal mol^-1^)** | $\int\mathbf{C}_{\mathbf{p}}\mathbf{dT}$  **(J mol^-1^)** | **E_ZPE_-TS**  **(kcal mol^-1^)** | **G**  **(Ha)** |
| --- | --- | --- | --- | --- | --- | --- |
| **H_2_(g)** | -1.1612253 | 8.306 | -9703.88805 | 2073.63325 | -1.398 | -1.163453188 |
| **CO_2_(g)** | -188.3997745 | 8.859 | -15776.3091 | 2790.38585 | -6.918 | -188.3942622 |
| **NiPc-TAB** | -2733.246748 | 378.512 | 45161.07865 | 51881.0815 | 333.351 | -2732.643542 |
| **[Ni-COO^-^]** | -2921.647876 | 388.68 | 56266.56985 | 55310.70095 | 332.414 | -2921.118133 |
| **[Ni-COOH]** | -2922.200738 | 394.55 | -50873.9308 | 55819.643 | 343.676 | -2921.653047 |
| **[Ni-CO]** | -2846.431761 | 383.355 | -46740.3792 | 53450.84125 | 336.615 | -2845.895323 |
| **[CO]** | -113.1844156 | 4.869 | -14113.8247 | 2077.87704 | -9.244 | -113.1991471 |
| **[H_2_O]** | -76.3586153 | 14.897 | -13894.3863 | 2388.1815 | 1.003 | -76.35701689 |
| **[H*]** | -2733.7801389 | 382.268 | -45373.95775 | 52650.3085 | 336.894 | -2733.243256 |

**Table S7** Calculated electronic energies (E_DFT_), zero-point vibrational energies (E_ZPE_), entropies (TS), thermal corrections ($\int C_{p}\mathrm{dT}$), and free energies (G) associated with Pc-THB catalyzed process

|  | **E_DFT_**  **(Ha)** | **E_ZPE_**  **(kcal mol^-1^)** | **-TS**  **(cal mol^-1^)** | $\int C_{p}\mathrm{dT}$  **(J mol^-1^)** | **E_ZPE_-TS**  **(kcal mol^-1^)** | **G**  **(Ha)** |
| --- | --- | --- | --- | --- | --- | --- |
| **H_2_(g)** | -1.1612253 | 8.306 | -9703.88805 | 2073.63325 | -1.398 | -1.163453188 |
| **CO_2_(g)** | -188.3997745 | 8.859 | -15776.3091 | 2790.38585 | -6.918 | -188.3942622 |
| **NiPc-THB** | -2891.8934661 | 310.833 | -44153.33165 | 50989.31485 | 266.68 | -2891.468485 |
| **[Ni-COO^-^]** | -3080.2940434 | 319.318 | -48877.81655 | 53453.82275 | 270.44 | -3079.86307 |
| **[Ni-COOH]** | -3080.8442696 | 327.219 | -50324.1422 | 54969.3192 | 276.894 | -3080.403011 |
| **[Ni-CO]** | -3005.0800678 | 316.768 | -52020.91385 | 53899.25885 | 264.747 | -3004.658167 |
| **[CO]** | -113.1844156 | 4.869 | -14113.8247 | 2077.87704 | -9.244 | -113.1991471 |
| **[H_2_O]** | -76.3586153 | 14.897 | -13894.3863 | 2388.1815 | 1.003 | -76.35701689 |
| **[H*]** | -2892.4299444 | 315.263 | -45914.20555 | 52032.24355 | 269.348 | -2892.000705 |

**Table S8** Calculated electronic energies (E_DFT_), zero-point vibrational energies (E_ZPE_), entropies (TS), thermal corrections ($\int C_{p}\mathrm{dT}$), and free energies (G) associated with NiPc-TTB catalyzed process

|  | **E_DFT_**  **(Ha)** | **E_ZPE_**  **(kcal mol^-1^)** | **-TS**  **(cal mol^-1^)** | $\int\mathbf{C}_{\mathbf{p}}\mathbf{dT}$  **(J mol^-1^)** | **E_ZPE_-TS**  **(kcal mol^-1^)** | **G**  **(Ha)** |
| --- | --- | --- | --- | --- | --- | --- |
| **H_2_(g)** | -1.1612253 | 8.306 | -9703.88805 | 2073.63325 | -1.398 | -1.163453188 |
| **CO_2_** | -188.3997745 | 8.859 | -15776.3091 | 2790.38585 | -6.918 | -188.3942622 |
| **NiPc-TTB** | -10949.7974501 | 617.842 | -105760.9606 | 108472.6349 | 512.081 | -10948.9814 |
| **[Ni-COO^-^]** | -11138.1976087 | 626.61 | -113079.3505 | 111584.1283 | 513.531 | -11137.37925 |
| **[Ni-COOH]** | -11138.7474524 | 632.777 | -112614.5347 | 112822.0471 | 520.162 | -11137.91852 |
| **[Ni-CO]** | -11062.9849335 | 623.72 | -111433.2644 | 111337.5582 | 512.286 | -11062.16855 |
| **[CO]** | -113.1844156 | 4.869 | -14113.8247 | 2077.87704 | -9.244 | -113.1991471 |
| **[H_2_O]** | -76.3586153 | 14.897 | -13894.3863 | 2388.1815 | 1.003 | -76.35701689 |
| **[H*]** | -10950.3079205 | 621.852 | -107176.28 | 109505.43 | 514.676 | -10949.48772 |

**Supplementary references**

1. Z. Iqbal, A. Lyubimtsev, M. Hanack, Synthesis of phthalonitriles using a palladium catalyst. Synlett 2287-2290 (2008). <https://doi.org/10.1055/s-2008-1078269>
2. S. Aldridge, R.J. Calder, M.H. Cunningham, K.M.A. Malik, J.W. Steed, Convenient syntheses, spectroscopic and structural characterisation of bi-functional boranes. J. Organomet. Chem. **614-615**, 188-194 (2000). <https://doi.org/10.1016/S0022-328X(00)00305-3>
3. H. Yuan, W. Gao, W. Xinhao, J. Ye, F. Ma, et al., Surface engineering of Pt aerogels by metal phthalocyanine to enhance the electrocatalytic property for oxygen reduction reaction. Mater. Today Energy **37**, 101379 (2023). <https://doi.org/10.1016/j.mtener.2023.101379>
4. H. Jia, Y. Yao, J. Zhao, Y. Gao, Z. Luo, et al., A novel two-dimensional nickel phthalocyanine-based metal–organic framework for highly efficient water oxidation catalysis. J. Mater. Chem. A **6**, 1188-1195 (2018). <http://dx.doi.org/10.1039/C7TA07978H>
5. W. Qi, W. Liu, B. Zhang, X. Gu, X. Guo, et al., Oxidative dehydrogenation on nanocarbon: identification and quantification of active sites by chemical titration. Angew. Chem. Int. Ed. **52**, 14224-14228 (2013). <https://doi.org/10.1002/anie.201306825>
6. S. Wu, G. Wen, R. Schlogl, D.S. Su, Carbon nanotubes oxidized by a green method as efficient metal-free catalysts for nitroarene reduction. Phys. Chem. Chem. Phys. **17**, 1567-1571 (2015). 10.1039/c4cp04658g
7. J. Tauc, R. Grigorovici, A. Vancu, Optical properties and electronic structure of amorphous germanium. Phys. Status. Solidi. B **15**, 627-637 (1966). <https://doi.org/10.1002/pssb.19660150224>
8. J. Tauc, R. Grigorovici, A. Vancu, Optical properties and electronic structure of amorphous germanium. Phys. Status. Solidi. B **15**, 627-637 (1966). <https://doi.org/10.1002/pssb.19660150224>
9. 9. T. Chen, J.-H. Dou, L. Yang, C. Sun, N.J. Libretto et al., Continuous electrical conductivity variation in M_3_(hexaiminotriphenylene)_2_ (M = Co, Ni, Cu) MOF alloys. J. Am. Chem. Soc. **142**, 12367-12373 (2020). <https://doi.org/10.1021/jacs.0c04458>
10. A. Mähringer, A.C. Jakowetz, J.M. Rotter, B.J. Bohn, J.K. Stolarczyk et al., Oriented thin films of electroactive triphenylene catecholate-based two-dimensional metal–organic frameworks. ACS Nano **13**, 6711-6719 (2019). <https://doi.org/10.1021/acsnano.9b01137>
11. L. Chen, J. Chen, W. Fu, J. Chen, D. Wang et al., Energy-efficient CO(_2_) conversion to multicarbon products at high rates on CuGa bimetallic catalyst. Nat. Commun. **15**, 7053 (2024). <https://doi.org/10.1038/s41467-024-51466-8>
12. M. Lu, M. Zhang, C.-G. Liu, J. Liu, L.-J. Shang et al., Stable dioxin-linked metallophthalocyanine covalent organic frameworks (COFs) as photo-coupled electrocatalysts for CO_2_ reduction. Angew. Chem. Int. Ed. **60**, 4864-4871 (2021). <https://doi.org/10.1002/anie.202011722>
13. T. Xie, S. Chen, Y. Yue, T. Sheng, N. Huang et al., Biomimetic phthalocyanine-based covalent organic frameworks with tunable pendant groups for electrocatalytic CO_2_ reduction. Angew. Chem. Int. Ed. **63**, e202411188 (2024). <https://doi.org/10.1002/anie.202411188>
14. X. Yang, X. Li, M. Liu, S. Yang, Q. Niu et al., Modulating electrochemical CO_2_ reduction performance via sulfur-containing linkages engineering in metallophthalocyanine based covalent organic frameworks. ACS Mater. Lett. **5**, 1611-1618 (2023). <https://doi.org/10.1021/acsmaterialslett.3c00168>
15. X. Yang, X. Li, M. Liu, S. Yang, Q. Xu et al., Quantitative construction of boronic-ester linkages in covalent organic frameworks for the carbon dioxide reduction. Angew. Chem. Int. Ed. **63**, e202317785 (2024). <https://doi.org/10.1002/anie.202317785>
16. B. Han, X. Ding, B. Yu, H. Wu, W. Zhou et al., Two-dimensional covalent organic frameworks with cobalt(II)-phthalocyanine sites for efficient electrocatalytic carbon dioxide reduction. J. Am. Chem. Soc. **143**, 7104-7113 (2021). <https://doi.org/10.1021/jacs.1c02145>
17. N. Huang, K.H. Lee, Y. Yue, X. Xu, S. Irle et al., A stable and conductive metallophthalocyanine framework for electrocatalytic carbon dioxide reduction in Water. Angew. Chem. Int. Ed. **59**, 16587-16593 (2020). <https://doi.org/10.1002/anie.202005274>
18. J. Yuan, S. Chen, Y. Zhang, R. Li, J. Zhang et al., Structural regulation of coupled phthalocyanine‐porphyrin covalent organic frameworks to highly active and selective electrocatalytic CO_2_ reduction. Adv. Mater. **34**, (2022). <http://doi.org/10.1002/adma.202203139>
19. S. Lin, C.S. Diercks, Y.-B. Zhang, N. Kornienko, E.M. Nichols et al., Covalent organic frameworks comprising cobalt porphyrins for catalytic CO_2_ reduction in water. Science **349**, 1208-1213 (2015). <https://doi.org/10.1126/science.aac8343>
20. R. Matheu, E. Gutierrez-Puebla, M.Á. Monge, C.S. Diercks, J. Kang et al., Three-Dimensional Phthalocyanine Metal-Catecholates for High Electrochemical Carbon Dioxide Reduction. J. Am. Chem. Soc. **141**, 17081-17085 (2019). <https://doi.org/10.1021/jacs.9b09298>
21. Y. Yue, P. Cai, K. Xu, H. Li, H. Chen et al., Stable bimetallic polyphthalocyanine covalent organic frameworks as superior electrocatalysts. J. Am. Chem. Soc. **143**, 18052-18060 (2021). <https://doi.org/10.1021/jacs.1c06238>
22. Q. Wu, R.-K. Xie, M.-J. Mao, G.-L. Chai, J.-D. Yi et al., Integration of strong electron transporter tetrathiafulvalene into metalloporphyrin-based covalent organic framework for highly efficient electroreduction of CO_2_. ACS Energy Letters **5**, 1005-1012 (2020). <https://doi.org/10.1021/acsenergylett.9b02756>
23. Y. Zhang, X. Zhang, L. Jiao, Z. Meng, H.-L. Jiang, Conductive covalent organic frameworks of polymetallophthalocyanines as a tunable platform for electrocatalysis. J. Am. Chem. Soc. **145**, 24230-24239 (2023). <https://doi.org/10.1021/jacs.3c08594>
24. N. Kornienko, Y. Zhao, C.S. Kley, C. Zhu, D. Kim et al., Metal–Organic Frameworks for Electrocatalytic Reduction of Carbon Dioxide. J. Am. Chem. Soc. **137**, 14129-14135 (2015). <https://doi.org/10.1021/jacs.5b08212>
25. J.-D. Yi, D.-H. Si, R. Xie, Q. Yin, M.-D. Zhang et al., Conductive two-dimensional phthalocyanine-based metal–organic framework nanosheets for efficient electroreduction of CO_2_. Angew. Chem. Int. Ed. **60**, 17108-17114 (2021). <https://doi.org/10.1002/anie.202104564>
26. L. Ye, J. Liu, Y. Gao, C. Gong, M. Addicoat et al., Highly oriented MOF thin film-based electrocatalytic device for the reduction of CO_2_ to CO exhibiting high faradaic efficiency. J. Mater. Chem. A **4**, 15320-15326 (2016). <http://dx.doi.org/10.1039/C6TA04801C>
27. B.-X. Dong, S.-L. Qian, F.-Y. Bu, Y.-C. Wu, L.-G. Feng et al., Electrochemical reduction of CO_2_ to CO by a heterogeneous catalyst of Fe–porphyrin-based metal–organic framework. ACS Appl. Energy Mater. **1**, 4662-4669 (2018). <https://doi.org/10.1021/acsaem.8b00797>
28. P.T. Smith, B.P. Benke, Z. Cao, Y. Kim, E.M. Nichols et al., Iron porphyrins embedded into a supramolecular porous organic cage for electrochemical CO_2_ reduction in water. Angew. Chem. Int. Ed. **57**, 9684-9688 (2018). <https://doi.org/10.1002/anie.201803873>
29. H. Zhong, M. Ghorbani-Asl, K.H. Ly, J. Zhang, J. Ge et al., Synergistic electroreduction of carbon dioxide to carbon monoxide on bimetallic layered conjugated metal-organic frameworks. Nat. Commun. **11**, 1409 (2020). <https://doi.org/10.1038/s41467-020-15141-y>
30. H.-J. Zhu, M. Lu, Y.-R. Wang, S.-J. Yao, M. Zhang et al., Efficient electron transmission in covalent organic framework nanosheets for highly active electrocatalytic carbon dioxide reduction. Nat. Commun. **11**, 497 (2020). <https://doi.org/10.1038/s41467-019-14237-4>
31. C. Costentin, S. Drouet, M. Robert, J.-M. Savéant, A local proton source enhances CO_2_ electroreduction to CO by a molecular Fe catalyst. Science **338**, 90-94 (2012). <https://doi.org/10.1126/science.1224581>
32. J. Shen, R. Kortlever, R. Kas, Y.Y. Birdja, O. Diaz-Morales et al., Electrocatalytic reduction of carbon dioxide to carbon monoxide and methane at an immobilized cobalt protoporphyrin. Nat. Commun. **6**, 8177 (2015). <https://doi.org/10.1038/ncomms9177>
33. N. Morlanés, K. Takanabe, V. Rodionov, Simultaneous reduction of CO_2_ and splitting of H_2_O by a single immobilized cobalt phthalocyanine electrocatalyst. ACS Catal. **6**, 3092-3095 (2016). <https://doi.org/10.1021/acscatal.6b00543>
34. Z. Weng, J. Jiang, Y. Wu, Z. Wu, X. Guo et al., Electrochemical CO_2_ reduction to hydrocarbons on a heterogeneous molecular Cu catalyst in aqueous solution. J. Am. Chem. Soc. **138**, 8076-8079 (2016). <https://doi.org/10.1021/jacs.6b04746>
35. Y. Wu, J. Jiang, Z. Weng, M. Wang, D.L.J. Broere et al., Electroreduction of CO_2_ catalyzed by a heterogenized Zn–porphyrin complex with a redox-innocent metal center. ACS Cent. Sci. **3**, 847-852 (2017). <https://doi.org/10.1021/acscentsci.7b00160>
36. N. Han, Y. Wang, L. Ma, J. Wen, J. Li et al., Supported cobalt polyphthalocyanine for high-performance electrocatalytic CO_2_ reduction. Chem **3**, 652-664 (2017). <http://doi.org/10.1016/j.chempr.2017.08.002>
37. S. Aoi, K. Mase, K. Ohkubo, S. Fukuzumi, Selective electrochemical reduction of CO_2_ to CO with a cobalt chlorin complex adsorbed on multi-walled carbon nanotubes in water. Chem. Commun. **51**, 10226-10228 (2015). <http://dx.doi.org/10.1039/C5CC03340C>
38. W.W. Kramer, C.C.L. McCrory, Polymer coordination promotes selective CO_2_ reduction by cobalt phthalocyanine. Chem. Sci. **7**, 2506-2515 (2016). <http://dx.doi.org/10.1039/C5SC04015A>
39. A. Maurin, M. Robert, Noncovalent immobilization of a molecular iron-based electrocatalyst on carbon electrodes for selective, efficient CO_2_-to-CO conversion in water. J. Am. Chem. Soc. **138**, 2492-2495 (2016). <https://doi.org/10.1021/jacs.5b12652>
40. X. Zhang, Z. Wu, X. Zhang, L. Li, Y. Li et al., Highly selective and active CO_2_ reduction electrocatalysts based on cobalt phthalocyanine/carbon nanotube hybrid structures. Nat. Commun. **8**, 14675 (2017). <https://doi.org/10.1038/ncomms14675>
41. X.-M. Hu, M.H. Rønne, S.U. Pedersen, T. Skrydstrup, K. Daasbjerg, Enhanced catalytic activity of cobalt porphyrin in CO_2_ electroreduction upon immobilization on carbon materials. Angew. Chem. Int. Ed. **56**, 6468-6472 (2017). <https://doi.org/10.1002/anie.201701104>
42. M. Zhu, J. Chen, L. Huang, R. Ye, J. Xu et al., Covalently grafting cobalt porphyrin onto carbon nanotubes for efficient CO_2_ electroreduction. Angew. Chem. Int. Ed. **58**, 6595-6599 (2019). <https://doi.org/10.1002/anie.201900499>
43. Y. Wu, Z. Jiang, X. Lu, Y. Liang, H. Wang, Domino electroreduction of CO_2_ to methanol on a molecular catalyst. Nature **575**, 639-642 (2019). <https://doi.org/10.1038/s41586-019-1760-8>
44. M. Wang, K. Torbensen, D. Salvatore, S. Ren, D. Joulié et al., CO_2_ electrochemical catalytic reduction with a highly active cobalt phthalocyanine. Nat. Commun. **10**, 3602 (2019). <https://doi.org/10.1038/s41467-019-11542-w>
45. T. Wang, L. Xu, Z. Chen, L. Guo, Y. Zhang et al., Central site regulation of cobalt porphyrin conjugated polymer to give highly active and selective CO_2_ reduction to CO in aqueous solution. Appl. Catal. B-Environ. **291**, 120128 (2021). <https://doi.org/10.1016/j.apcatb.2021.120128>
